# Induction of Strain-Transcending Antibodies Against Group A PfEMP1 Surface Antigens from Virulent Malaria Parasites

**DOI:** 10.1371/journal.ppat.1002665

**Published:** 2012-04-19

**Authors:** Ashfaq Ghumra, Jean-Philippe Semblat, Ricardo Ataide, Carolyne Kifude, Yvonne Adams, Antoine Claessens, Damian N. Anong, Peter C. Bull, Clare Fennell, Monica Arman, Alfred Amambua-Ngwa, Michael Walther, David J. Conway, Lalla Kassambara, Ogobara K. Doumbo, Ahmed Raza, J. Alexandra Rowe

**Affiliations:** 1 Centre for Immunity, Infection and Evolution, Institute of Immunology and Infection Research, School of Biological Sciences, University of Edinburgh, Edinburgh, United Kingdom; 2 Kenya Medical Research Institute-Wellcome Trust Research Programme, Kilifi, Kenya; 3 Biotechnology Unit, Faculty of Science, University of Buea, Buea, Cameroon; 4 Medical Research Council Laboratories, Fajara, Banjul, The Gambia; 5 Malaria Research and Training Centre, University of Bamako, Bamako, Mali; Seattle Biomedical Research Institute, United States of America

## Abstract

Sequence diversity in pathogen antigens is an obstacle to the development of interventions against many infectious diseases. In malaria caused by *Plasmodium falciparum*, the PfEMP1 family of variant surface antigens encoded by *var* genes are adhesion molecules that play a pivotal role in malaria pathogenesis and clinical disease. PfEMP1 is a major target of protective immunity, however, development of drugs or vaccines based on PfEMP1 is problematic due to extensive sequence diversity within the PfEMP1 family. Here we identified the PfEMP1 variants transcribed by *P. falciparum* strains selected for a virulence-associated adhesion phenotype (IgM-positive rosetting). The parasites transcribed a subset of Group A PfEMP1 variants characterised by an unusual PfEMP1 architecture and a distinct N-terminal domain (either DBLα1.5 or DBLα1.8 type). Antibodies raised in rabbits against the N-terminal domains showed functional activity (surface reactivity with live infected erythrocytes (IEs), rosette inhibition and induction of phagocytosis of IEs) down to low concentrations (<10 µg/ml of total IgG) against homologous parasites. Furthermore, the antibodies showed broad cross-reactivity against heterologous parasite strains with the same rosetting phenotype, including clinical isolates from four sub-Saharan African countries that showed surface reactivity with either DBLα1.5 antibodies (variant HB3var6) or DBLα1.8 antibodies (variant TM284var1). These data show that parasites with a virulence-associated adhesion phenotype share IE surface epitopes that can be targeted by strain-transcending antibodies to PfEMP1. The existence of shared surface epitopes amongst functionally similar disease-associated *P. falciparum* parasite isolates suggests that development of therapeutic interventions to prevent severe malaria is a realistic goal.

## Author Summary

Malaria remains one of the world's most deadly diseases. Life-threatening malaria is linked to a process called rosetting, in which malaria parasite-infected red blood cells bind to uninfected red cells to form aggregates that block blood flow in vital organs such as the brain. Current efforts to develop drugs or vaccines against rosetting are hindered by variation in the parasite rosette-mediating proteins, found on the surface of infected red cells. We studied these parasite-derived surface proteins and discovered that although they are variable, they share some common features. We raised antibodies against the rosette-mediating proteins, and found that they cross-reacted with multiple rosetting parasite strains from different countries around the world, including samples collected directly from African children with severe malaria. These findings provide new insights into malaria parasite interactions with human cells, and provide proof of principle that variable parasite molecules from virulent malaria parasites can induce strain-transcending antibodies. Hence, this work provides the foundation for the development of new therapies to treat or prevent life-threatening malaria.

## Introduction

The design of new drugs and vaccines against many infectious diseases is hindered by sequence diversity in key pathogen antigens [1]. This is a particular problem in the deadliest form of human malaria caused by *P. falciparum*, in which important targets of protective immunity are highly variable antigens (PfEMP1 variants, encoded by *var* genes) expressed on the surface of IEs [2]. Every *P. falciparum* isolate has 50–60 diverse PfEMP1 variants, and the PfEMP1 repertoires of different isolates are largely non-overlapping [3]–[6]. PfEMP1 variants are expressed in a mutually exclusive fashion, and transcriptional switching from one *var* gene to another results in antigenic variation of *P. falciparum* IEs [7]. PfEMP1 variants sampled from broad global parasite populations show essentially unlimited amino acid sequence diversity [5], [8], making PfEMP1 an extremely challenging therapeutic target [9], [10]. Surface-reactive antibodies to PfEMP1 on live IEs that occur after natural infections [11], [12] or after immunization with recombinant PfEMP1 domains [12], [13] are predominantly variant- and strain-specific, as expected for highly variable parasite antigens. However, children living in endemic areas develop antibodies during the first few years of life that protect against life-threatening malaria [14] suggesting that strain-transcending antibody responses may occur [15], or that the parasites that cause severe malaria are of restricted antigenic types [16], [17]. Antigenically-restricted subsets of parasite surface antigens that induce strain-transcending antibodies have not yet been identified.

In addition to their role in immunity and immune evasion, PfEMP1 variants are adhesion molecules that mediate interactions with a variety of human cell types and surface receptors [18], [19]. Three major PfEMP1 families (A, B and C, based on conserved upstream sequence and genomic location) differ in their adhesive function [18]. Group B and C variants (approximately 40–50 variants per haploid parasite genome) bind to the endothelial protein and scavenger receptor CD36 [20], [21]. In contrast, Group A variants (approximately 10 variants per haploid parasite genome) do not bind CD36 [20], [21]. The binding functions of most Group A variants are currently unknown, except for several examples of Group A variants that mediate rosetting [12], [13], [22], [23], an adhesion phenotype in which IEs bind to uninfected Es [24]. The fact that different antigenic forms of PfEMP1 mediate different binding phenotypes means that transcriptional switching of *var* genes not only results in antigenic variation, but can also result in alteration of the adhesion phenotype of IEs [25] and the propensity to cause disease. Several studies have examined the link between *var* gene transcription and clinical disease, and most show that transcription of Group A *var* genes is linked to severe malaria in a variety of geographical settings [26]–[29] and laboratory experiments [30], whereas transcription of B and C *var* genes occurs in less virulent infections causing uncomplicated disease [26]–[29].

Rosetting is currently the adhesion phenotype mostly clearly linked to parasite virulence, being associated with life-threatening malaria in African children [31]–[35] and high parasite burden in a primate malaria model [36]. Rosetting causes pathological obstruction to microvascular blood flow [37] and human erythrocyte polymorphisms that reduce the ability of *P. falciparum* to form rosettes confer substantial protection against severe malaria [38], [39]. *P. falciparum* rosetting parasites can be divided into two distinct phenotypes: those that bind IgM natural antibodies (“non-immune” IgM) from normal human plasma/serum onto the surface of IEs (here called IgM-positive rosetting) [40], [41] and those that do not (IgM-negative rosetting). Non-immune IgM-binding is thought to strengthen the adhesion interactions between infected and uninfected Es in rosettes [40], [42], [43], and may also play a role in immune evasion by masking key epitopes [44]. Previous studies of PfEMP1 and rosetting have focussed on parasites with the IgM-negative phenotype [12], [13], [22], [23], [45]. Detailed examination of IgM-positive rosetting parasites has been neglected to date, despite the clinical importance of this phenotype. A previous study of 57 clinical isolates from Kenyan children with severe and uncomplicated malaria found that 46 isolates formed rosettes (with rosette frequency ranging from 1% to 79%) and all rosetting isolates showed IgM-binding [41]. There was a strong positive correlation between rosette frequency and the percentage of IgM-positive IEs (ρ = 0.804, p<0.001, Spearman correlation). IgM-positive IEs were not seen in parasite strains showing other common adhesion phenotypes such as CD36 binding, ICAM-1 binding or platelet-mediated clumping [41]. IgM-positive IEs are also found in chondroitin sulfate A-binding parasite strains linked to pregnancy malaria [46], however parasites with this phenotype are rare in children [47]. Therefore in malaria infections of young children, IgM-binding and rosetting are linked phenotypes and are associated with severe disease [41].

Here we examine representatives from both major rosetting phenotypes to identify PfEMP1 variants responsible for rosetting and to investigate the hypothesis that PfEMP1 variants from *P. falciparum* parasites with a shared virulence-associated adhesion phenotype might share surface epitopes. We found that IgM-positive rosetting parasites transcribe a subset of PfEMP1 variants and that immunization with the N-terminal domain of these variants generates strain-transcending antibodies that recognise geographically diverse IgM-positive rosetting strains.

## Results

### Identification of PfEMP1 variants transcribed by rosetting parasites

**Figure 1:**
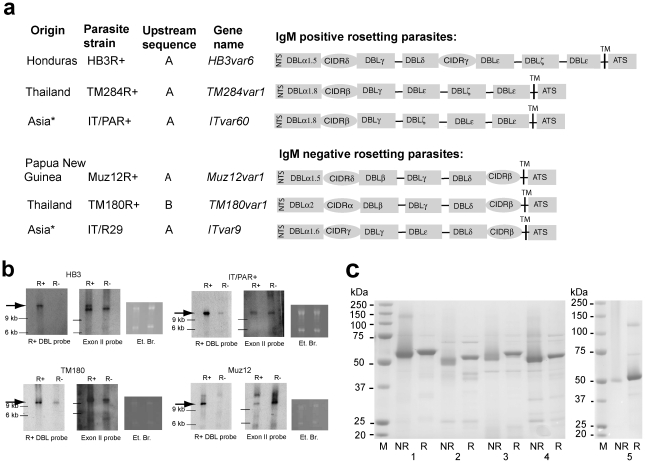
Identification of key surface antigens (Group A PfEMP1 variants) of *P. falciparum* rosetting parasites and production of recombinant proteins for immunization. a) PfEMP1 domain architecture of the predominantly expressed variants from *P. falciparum* rosetting laboratory strains. The previously described rosetting variant ITvar9 [13], [22], [45] is shown for comparison. Domain types are based on conserved motifs [6], [51]. NTS: N-Terminal Segment; DBL: Duffy Binding Like; CIDR: Cysteine-rich InterDomain Region; ATS: Acidic Terminal Segment; TM: TransMembrane region. *The IT isolate was originally from Brazil, however following cross-contamination of parasite cultures in the early1980s, current IT/FCR3 strains are thought to be of South-East Asian origin [88]. The Genbank accession numbers for these sequences are Y13402 (*ITvar9/R29var1*), EF158099 (*ITvar60*), JQ684046 (*TM284var1*), JQ684047 (*TM180var1*) and JQ684048 (*Muz12var1*). The *HB3var6* sequence can be obtained from http://www.broadinstitute.org/annotation/ genome/plasmodium_falciparum_spp/MultiHome.html gene reference PFHG_02274.1. b) Northern blots of RNA from isogenic rosetting (R+) and non-rosetting (R−) parasites probed with a PfEMP1 domain from the rosette-specific variant for each strain (R+ DBL probe, high stringency) and with an Exon II probe (moderate stringency), which detects all *var* genes [50]. Arrows indicate the major rosette-specific *var* gene transcript in each strain. Equal loading of R+ and R− RNA was confirmed by staining with ethidium bromide (Et Br). c) Production of recombinant NTS-DBLα domains in *E. coli* to immunize rabbits. 1: TM180var1, 2: Muz12var1, 3:TM284var1, 4: ITvar60, 5:HB3var6. M: molecular weight marker; R: reduced; NR: non-reduced.

To identify the key surface antigens of rosetting parasites, five *P. falciparum* laboratory strains originating from different countries were grown *in vitro* and selected for the rosetting phenotype. Three IgM-positive (HB3R+, TM284R+ and IT/PAR+) and two IgM-negative (Muz12R+ and TM180R+) rosetting strains were studied (see “Materials and Methods” for full details of parasite strains). For each strain, isogenic rosette positive (R+) and rosette negative (R−) populations were selected in parallel [22], [48], and their *var* gene transcription profiles examined by analysis of short PfEMP1 sequence tags [27]. The rosette-specific variant in each strain was identified as the predominant *var* gene transcribed by the rosetting population (comprising between one third to one half of all the *var* gene sequences detected) that was absent/rare in the non-rosetting population (an example is shown in Table S1). The full-length sequence of each predominant rosette-specific *var* gene was obtained from the sequence tag as described in the Materials and Methods. The rosetting variants were mostly Group A (Figure 1a), defined by the presence of a conserved upstream sequence (UpsA) and a characteristic N-terminal domain type (called DBLα1 or “Cys2”) that is associated with severe malaria [20], [27], [29]. The variants from the IgM-positive rosetting parasites form a distinct subset that share an unusual PfEMP1 architecture, containing a triplet of domains that occur rarely in PfEMP1 (DBLε and DBLζ) [6] preceding the transmembrane region. The binding site for non-immune IgM lies within these DBLε/ζ domains [49], [50](AG and JAR, unpublished data). The IgM-binding domain triplet is linked via at least one other domain (DBLγ) to a typical Group A PfEMP1 head-structure [18], [20], [51] (Figure 1a). DBLα domains from Group A PfEMP1 variants fall into eight subclasses (DBLα1.1 to DBLα1.8) based on sequence homology [6]. The rosetting variants described previously (ITvar9 [22], Palo Alto varO [23] and PF13_0003 [12]) are all of the DBLα1.6 subclass. The rosette-specific variants identified here are DBLα1.5 (HB3var6 and Muz12var1), DBLα1.8 (TM284var1 and ITvar60) or DBLα2 (a Group B type, TM180var1) [6].

Despite the observed similarities in PfEMP1 architecture, there was considerable sequence diversity amongst the rosette-specific variants from different parasite strains, with the rosette-mediating domain (NTS-DBLα) [12], [22], [23] showing pair-wise amino acid identities of between 38.9% (ITvar60:TM180var1) and 62.6% (ITvar60:TM284var1) (Table S2 and Figure S1). The other extracellular domains from the rosetting variants do not show high levels of amino acid identity apart from the first CIDR domain of TM284var1 and ITvar60 (82.2%) and the first CIDR domain of HB3var6 and Muz12var1 (81.1%; see Tables S2, S3, S4, S5, S6, S7 for pair-wise amino acid identities for all domain types).

Northern blots were carried out to determine whether rosetting parasite-specific PfEMP1 variants had been identified. For each parasite strain, a specific PfEMP1 domain from the rosetting-associated variant identified above was used to probe RNA from isogenic pairs of rosetting and non-rosetting parasites. The rosetting-associated PfEMP1 probe detected a transcript in rosetting parasites (arrowed) that was absent/weak in isogenic non-rosetting parasites (Figure 1b; shown previously for TM284 [50]). The presence of other transcribed *var* genes in the non-rosetting parasites was shown using an Exon II probe that identifies all *var* genes (Figure 1b). These data show that the transcriptional profiling experiments correctly identified full-length *var* genes whose transcription is specific to rosette-selected parasites.

In order to raise antibodies against the rosetting PfEMP1 variants, the N-terminal NTS-DBLα region of each rosetting parasite-specific variant was expressed as a recombinant protein in *E. coli* [13], with a shift in mobility of the recombinant proteins upon reduction showing the presence of disulfide bonds in these cysteine-rich proteins (Figure 1c). NTS-DBLα was chosen because it is the domain that binds erythrocytes to bring about rosetting [22], [23], and variant-specific antibodies to this region were the most effective in inhibiting rosetting in previous studies [13], [23].

### Polyclonal antibodies against PfEMP1 recognize the surface of live IEs of homologous *P. falciparum* rosetting strains

The recombinant proteins were used to immunize rabbits [13], to raise polyclonal antibodies to the PfEMP1 variants from each of the five different *P. falciparum* rosetting strains. Two rabbits were immunised per antigen and the resulting antisera were tested against the antigen used for immunization in an ELISA. Very similar responses were obtained from each pair of rabbits, with ELISA values (50% of maximum titre) of >1/40,000 (HB3var6 and Muz12var1) or >1/100,000 (TM284var1, ITvar60 and TM180var1).

**Figure 2:**
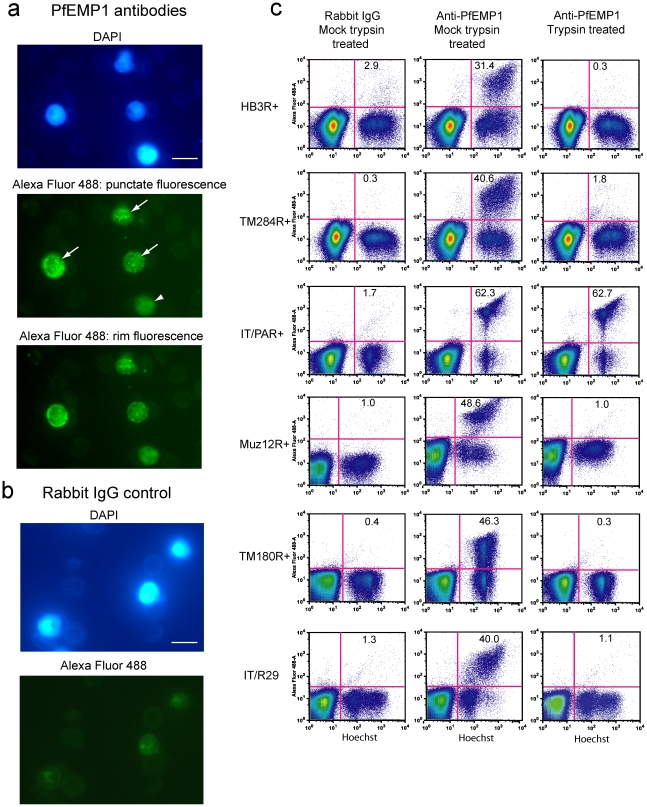
Polyclonal antibodies to PfEMP1 recognize the surface of homologous live infected erythrocytes (IEs). a) Live cell ImmunoFluorescence Assay (IFA) with antibodies to HB3var6 (1/50 dilution) tested on the homologous parasite (HB3R+). DAPI staining (1 µg/ml) shows the position of IEs (upper panel; scale bar 10 µm). PfEMP1 antibody is detected by highly cross-absorbed Alex Fluor 488-conjugated anti-rabbit IgG (1/500 dilution, middle and lower panels). Specific staining of IEs is seen as punctate fluorescence over the whole IE surface (middle panel, white arrows). Unstained IEs show pale smooth background fluorescence (middle panel, white arrowhead). If the plane of focus is adjusted, stained IEs show mainly rim fluorescence (lower panel). Rosettes are not seen in these images because they are disrupted by the PfEMP1 antibodies. b) IFA with antibodies from a non-immunized control rabbit (1/50 dilution) tested on HB3R+ parasite culture. Upper panel: DAPI staining shows the position of IEs (scale bar 10 µm). Lower panel: highly cross-absorbed Alex Fluor 488-conjugated anti-rabbit IgG gives no specific staining on IEs. Camera exposure settings and image handling for Alexa Fluor 488 images were identical for PfEMP1 antibody and control pictures. c) Flow cytometry of live IEs of *P. falciparum* rosetting strains stained with homologous PfEMP1 antibodies (HB3R+ parasites with HB3var6 antibodies; TM284R+ parasites with TM284var1 antibodies; IT/PAR+ parasites with ITvar60 antibodies; Muz12R+ parasites with Muz12var1 antibodies; TM180R+ parasites with TM180var1 antibodies; IT/R29 parasites with ITvar9 antibodies). Negative control rabbit IgG from a non-immunized rabbit (left column) and PfEMP1 antibodies (middle column) were tested at 100 µg/ml of total IgG. IEs were stained with Hoechst and rabbit IgG bound to the surface of erythrocytes was detected with highly cross-absorbed Alex Fluor 488-conjugated anti-rabbit IgG at 1/500 dilution. The percentage of Hoechst-stained IEs that were stained with Alexa Fluor 488 is shown in the upper right quadrant. The IE molecules recognised by PfEMP1 antibodies were sensitive to trypsin (right column) (10 µg/ml trypsin for 5 mins at room temperature (RT), followed by 1 mg/ml of trypsin inhibitor for 5 mins at RT), except for parasite strain IT/PAR+, in which the surface molecules recognized by ITvar60 antibodies were trypsin-resistant, even at 1 mg/ml of trypsin. Rabbit polyclonal antibodies to ITvar9 expressed by IT/R29 rosetting parasites have been reported previously [13], and are included here in all figures for comparison with the newly generated antibodies to the five other rosetting strains.

To determine if the antibodies recognised native PfEMP1 on the surface of live IEs, they were tested by ImmunoFluorescence Assay (IFA) and flow cytometry against homologous parasites (defined here as meaning antibodies against a particular PfEMP1 variant being tested against the parasite strain from which that variant was identified as the predominant PfEMP1). The antisera to each of the five variants gave punctate surface fluorescence of homologous IEs that is characteristic of PfEMP1 antibody staining [13], [52]–[54] (Figure 2a middle panel). Between 30–75% of IEs in each culture showed punctate staining, similar to the rosette frequency in these laboratory strains (which varies from cycle to cycle due to *var* gene switching and frequency of rosette selection) (Table S8). Depending on the plane of focus, the staining of live IEs in IFA wet preparations can also be seen as rim fluorescence as described in some previous publications [23] (Figure 2a lower panel). The pre-immune serum from each rabbit and serum from a non-immunized control rabbit did not show punctate staining of IEs by IFA. These negative controls show faint, smooth background fluorescence over both infected and uninfected Es by fluorescence microscopy (Figure 2b, lower panel). Antibodies to a non-rosetting Group A PfEMP1 variant HB3var3 (a variant transcribed by non-rosetting parasites that bind to brain endothelial cells, Claessens and Rowe *et al*, submitted) gave the same negative IFA appearance as control non-immunized rabbit serum shown in Figure 2b. For all immunizations, the antisera from the two rabbits per antigen gave similar results. For each antigen, the antiserum giving the brightest IFA signal at 1/50 dilution was chosen for purification of total IgG for subsequent experiments.

By flow cytometry using homologous antibody/parasite combinations, dot plots showed a population of IEs that were surface stained with PfEMP1 antibodies (Figure 2c, middle column, upper right quadrants). IgG from a control non-immunized rabbit did not stain IEs (Figure 2c, left column). One of the features of the PfEMP1 family is that most variants show unusual sensitivity to trypsin and can be cleaved from the surface of IEs by very low concentrations of protease [55]. To determine whether the antibodies raised to PfEMP1 NTS-DBLα domains were recognising PfEMP1-like molecules on the surface of live IEs, we carried out immunofluorescent staining and flow cytometry after treatment of live IEs with a low concentration of trypsin. We found that for parasite strains HB3R+, TM284R+, Muz12R+, TM180R+ and IT/R29, the staining with homologous PfEMP1 antibodies was abolished by mild trypsinisation (Figure 2c, right column), consistent with recognition of PfEMP1. For parasite strain IT/PAR+ however, antibodies to ITvar60 detected IE surface molecules that were resistant to proteolytic cleavage, even up to 1 mg/ml of trypsin (Figure 2c, right column). This suggests either that the ITvar60 PfEMP1 variant is trypsin-resistant or that the antibodies to ITvar60 are recognising other (non-PfEMP1, trypsin-resistant) molecules on the IE surface. Western blots to investigate these possibilities showed that IT/PAR+ parasites do express a trypsin-resistant PfEMP1 variant (Figure S2a and Text S1), and that the rabbit polyclonal antibodies to ITvar60 recognise high molecular weight parasite-specific trypsin-resistant molecules, and no other parasite-specific molecules were identified (Figure S2c and Text S1).

**Figure 3 ppat-1002665-g003:**
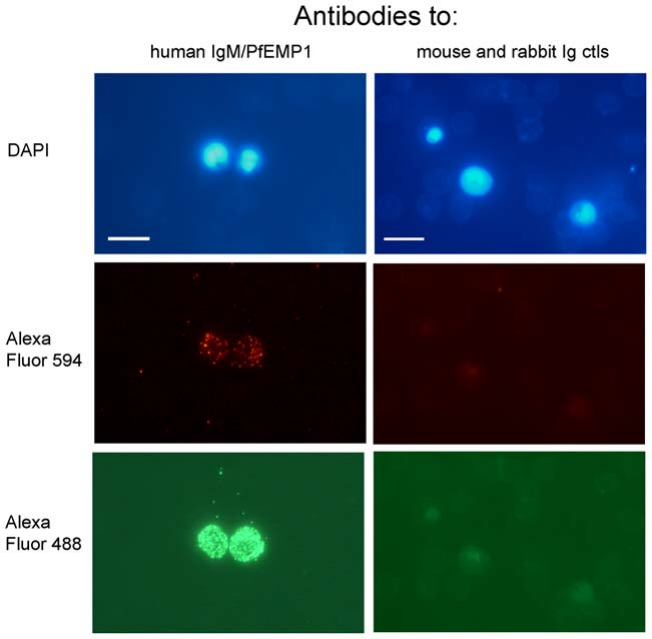
Polyclonal antibodies to PfEMP1 recognize IgM-positive IEs. HB3R+ live IEs were stained with a mixture of mouse mAb anti-human IgM (1/500 dilution) and rabbit polyclonal HB3var6 NTS-DBLα antibodies (20 µg/ml) (left column) or a mixture of mouse IgG isotype control and non-immunized rabbit IgG control (right column). Secondary incubation was with a mixture of Alexa 488 conjugated anti-rabbit IgG (1/1000) and Alexa 594-conjugated anti-mouse IgG (1/1000). IEs were stained with DAPI (1 µg/ml; scale bar 10 µm). The PfEMP1 antibodies (left column, bottom panel) stained IEs that were also positive for human IgM (left column, middle panel). Camera exposure settings were identical for PfEMP1 antibodies and controls except for human IgM/PfEMP1 with Alexa Fluor 488 which was taken at a shorter exposure setting (20 msecs) than the control (200 msecs), due to the brightness of the signal.

For the IgM-positive rosetting strains (HB3R+, TM284R+ and IT/PAR+), we tested whether the homologous PfEMP1 antibodies recognized the IgM-positive IEs by dual colour IFA. For all three strains, the same individual IEs were stained with anti-human IgM (red) and anti-PfEMP1 (green) (HB3R+ parasites shown in Figure 3 and TM284R+ parasites shown in Figure S3). For all three strains, 94–100% of the IEs that stained with the PfEMP1 antibodies were IgM-positive. Similarly, 91–100% of the IgM-positive IEs were positive with the PfEMP1 antibodies. Secondary antibody-only controls (not shown) and species-specific Ig controls (Figure 3, right column) were negative by IFA. In addition, combinations of rabbit PfEMP1 antibodies with anti-mouse secondary and mouse human IgM antibody with anti-rabbit secondary were also negative (Figure S3b), ruling out the possibility of non-specific binding of the Alexa Fluor-conjugated secondary antibodies. Additional positive controls (mouse anti-human IgM alone with anti-mouse secondary and rabbit PfEMP1 antibody alone with anti-rabbit secondary) showed the expected positive staining (not shown). The IgM staining did not differ in the presence or absence of the PfEMP1 antibodies (not shown), suggesting that the binding of antibodies to the N-terminal domains of PfEMP1 does not interfere with IgM-binding towards the C-terminus of the molecule [49], [50].

These experiments show that for parasite strains HB3R+, TM284R+ and IT/PAR+, the homologous PfEMP1 antibodies are specifically recognising the IgM-binding IE population, which are the rosette-forming cells ([41] and Table S9). This confirms that transcriptional profiling correctly identified the predominant PfEMP1 variant (Figure 1) from the IgM-positive rosette-selected parasite culture of each strain.

### Polyclonal antibodies against PfEMP1 recognize the surface of live IEs of heterologous *P. falciparum* rosetting strains

**Figure 4:**
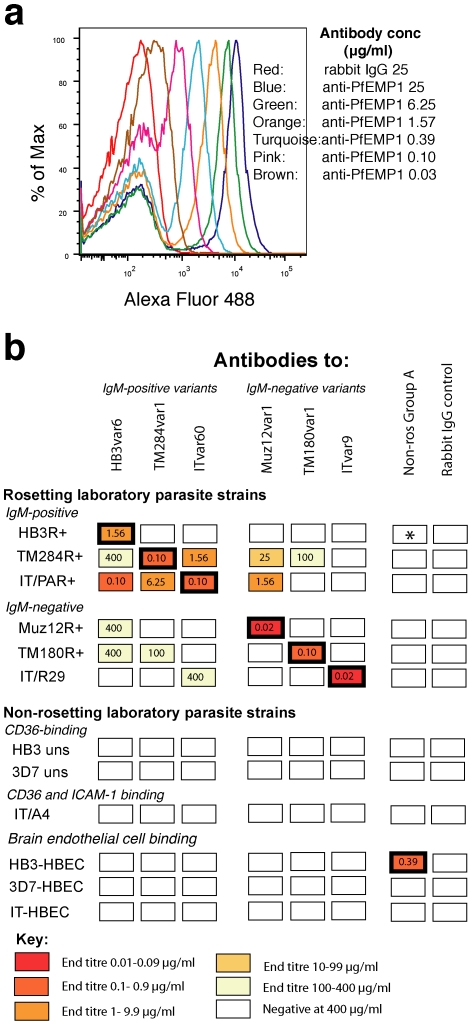
Polyclonal antibodies to PfEMP1 recognize the surface of homologous and heterologous live IEs. a) An example of the determination of the immunofluorescence end titre. Flow cytometry histograms showing the titration of antibodies to ITvar60 against IT/PAR+ parasites, compared to a non-immunized rabbit IgG control. The end titre (defined here as the lowest concentration of antibody giving surface staining above rabbit IgG background levels of more than 50% of the positive IE subpopulation) was 0.1 µg/ml. b) PfEMP1 antibodies (four-fold dilutions of total IgG starting at 400 µg/ml) were tested in IFA or flow cytometry against *P. falciparum* laboratory strains with various different adhesion phenotypes as indicated. The end titre for each antibody/parasite combination is shown inside each rectangle, with homologous antibody/parasite combinations being outlined in bold. Negative controls were non-immunized rabbit IgG control, and antibodies against NTS-DBLα from a non-rosetting Group A PfEMP1 variant (Non-ros Group A: HB3var3, expressed by HB3-HBEC which are non-rosetting parasites selected for binding to human brain endothelial cells [83]). *The HB3R+ parasites contain a subpopulation of non-rosetting HB3var3-expressing IEs (Table S1) that are distinct from the IgM-positive HB3var6-expressing rosetting IEs.

To determine whether the PfEMP1 antibodies show surface reactivity when tested against heterologous parasite strains, we carried out live IE IFA and flow cytometry with heterologous antibody/parasite combinations, and assessed the end titre of any combinations showing positive surface fluorescence. The end titres of homologous antibody/parasite combinations were also determined for comparison. End titres were determined using four-fold dilutions of antibody and are defined here as the lowest concentration giving surface staining of more than 50% of the positive subpopulation (Figure 4a, shown for IT/PAR+ parasites and ITvar60 antibodies).

We found that antibodies to PfEMP1 showed specific surface reactivity against homologous parasites down to low concentrations (end titres of <2 µg/ml of total IgG, Figure 4b, rectangles in bold). Against heterologous parasite strains, several of the PfEMP1 antibodies also showed good surface staining of other rosetting strains down to low concentrations (<10 µg/ml of total IgG, Figure 4b). This was especially marked with antibodies to the PfEMP1 variants from IgM-positive rosetting parasites. For example, ITvar60 antibodies stained TM284R+ parasites down to low concentrations and vice versa (TM284var1 antibodies stained IT/PAR+ parasites). IT/PAR+ parasites were also stained with low concentrations of HB3var6 antibodies (Figure 4b). Within each parasite population, dual colour IFA showed that the heterologous PfEMP1 antibodies recognised the IgM-positive IE population (shown for TM284R+ parasites, Figure S3). Furthermore, heterologous antibodies recognised trypsin-sensitive surface molecules on parasite strain TM284R+, consistent with binding to PfEMP1 (Figure S4a). While for parasite strain IT/PAR+, heterologous antibodies recognised trypsin-resistant molecules (Figure S4b) as seen with the homologous antibody (Figure 2). The antibodies raised to the PfEMP1 variants from IgM-positive rosetting parasites also showed some reactivity with the IgM-negative rosetting strains (Muz12R+, TM180R+ and IT/R29), although high concentrations were required (100–400 µg/ml of total IgG, Figure 4b). These concentrations still represent a considerable dilution of whole serum (equivalent to 1/100 to1/25 dilution) therefore they are potentially relevant *in vivo*. Antibodies raised to the PfEMP1 variants from IgM-negative rosetting parasites were predominantly variant- and strain-specific and showed only limited surface reactivity with the other rosetting laboratory strains (Figure 4b), consistent with previous data [12], [13].

The PfEMP1 antibodies were also tested for surface reactivity against parasite lines showing other adhesion phenotypes. We found that antibodies raised against rosetting PfEMP1 variants did not recognise parasites showing other adhesion phenotypes (Figure 4b), including binding to CD36 or ICAM-1 (parasites expressing Group B and C *var* genes) or binding to brain endothelial cells (parasites expressing an alternative sub-set of group A and B/A *var* genes, Claessens and Rowe *et al*, submitted).

Taken together, the above data show that polyclonal antibodies generated against PfEMP1 variants from IgM-positive rosetting strains have strain-transcending properties, as they show surface reactivity with heterologous rosetting strains, especially those showing IgM-positive rosetting. This suggests shared surface epitopes amongst heterologous rosetting PfEMP1 variants.

We examined whether similar patterns of variant-specific and cross-reactive antibody responses to those shown above were found when each total IgG preparation was tested in an ELISA against the panel of NTS-DBLα recombinant proteins used for immunization. We found that although each antibody showed high ELISA O.D. readings against the homologous immunizing antigen, they also showed widespread recognition of other DBL domains using this method (Figure S5). These data confirm earlier findings of Vigan-Womas *et al* [12] who showed that PfEMP1 antibody recognition of DBL domains by ELISA does not successfully predict surface reactivity with live IEs.

### Strain-transcending polyclonal PfEMP1 antibodies are functionally active

**Figure 5 ppat-1002665-g005:**
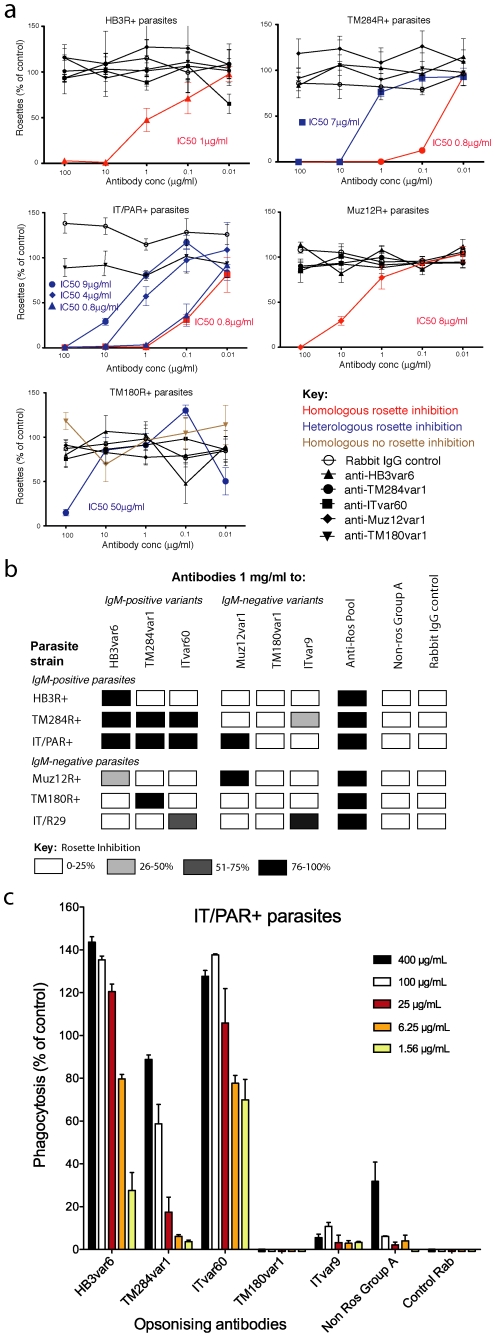
Polyclonal antibodies to PfEMP1 inhibit rosetting and induce phagocytosis of heterologous rosetting laboratory strains. a) Rosette inhibition assays to determine the dose-dependent effects of PfEMP1 antibodies on homologous and heterologous rosetting laboratory strains. Data are compared to a control with no added antibody, which contained at least 40% of IEs in rosettes. Mean and standard deviation of triplicate values are shown. IC50: concentration of antibody giving 50% rosette inhibition. b) Rosette inhibition assay as above with 1 mg/ml of antibody, except for the Anti-Ros pool which consisted of a mixture of 0.1 mg/ml of each antibody (to HB3var6, TM284var1, ITvar60, Muz12var1, TM180var1 and ITvar9). Controls are as for Figure 4b. c) Phagocytosis assay of opsonised IT/PAR+ IEs co-incubated with the monocytic cell line Thp-1 [13]. Data are shown as percentage of the positive control opsonised with a rabbit anti-human erythrocyte antibody. Both homologous and heterologous antibodies induce phagocytosis of IT/PAR+ IEs. Control Rab: negative control of IgG from a non-immunized rabbit.

Surface recognition of live IEs by antibodies *in vivo* is likely to lead to parasite clearance via effector mechanisms such as phagocytosis or complement-mediated lysis [14]. Rosette-inhibition may also be desirable *in vivo* to prevent pathological microvascular obstruction. We therefore examined whether the surface reactivity by homologous and heterologous PfEMP1 antibodies shown in Figure 4b, translated into demonstrable effector functions. The PfEMP1 antibodies showed potent rosette-inhibition against homologous parasite strains with 50% inhibitory concentrations (IC50) for rosetting between 0.8–8 µg/ml of total IgG (Figure 5a, red curves), except for TM180R+, which was not inhibited (Figure 5a, brown curve) despite good surface reactivity (Figure 4b). Parasite strains TM284R+, IT/PAR+ and TM180R+ all showed rosette inhibition by heterologous antibodies (Figure 5a, blue curves). Two repeated experiments with TM180R+ confirmed the lack of rosette inhibition by homologous antibody and successful rosette inhibition by heterologous TM284var1 antibody. At a higher concentration (1 mg/ml of total IgG, equivalent to 1/10 dilution of serum) the cross-reactivity in rosette inhibition was even more marked, with all strains being inhibited by antibodies to at least one of the IgM-positive rosetting PfEMP1 variants (Figure 5b). These concentrations are equivalent to those seen with naturally-acquired rosette-disrupting antibodies in malaria-exposed patients which show activity at 1/10 or 1/5 dilution [56].

The antibodies to PfEMP1 variants from IgM-positive rosetting parasites were also shown to have cross-reactive opsonising effects, by inducing the phagocytosis of homologous and heterologous IEs (Figure 5c and Figure S6). In contrast, antibodies to PfEMP1 variants from IgM-negative rosetting parasites only effectively opsonised homologous parasites (Figure 5c and Figure S6).

### Polyclonal antibodies against PfEMP1 show surface-reactivity and rosette inhibition against *P. falciparum* clinical isolates

**Figure 6 ppat-1002665-g006:**
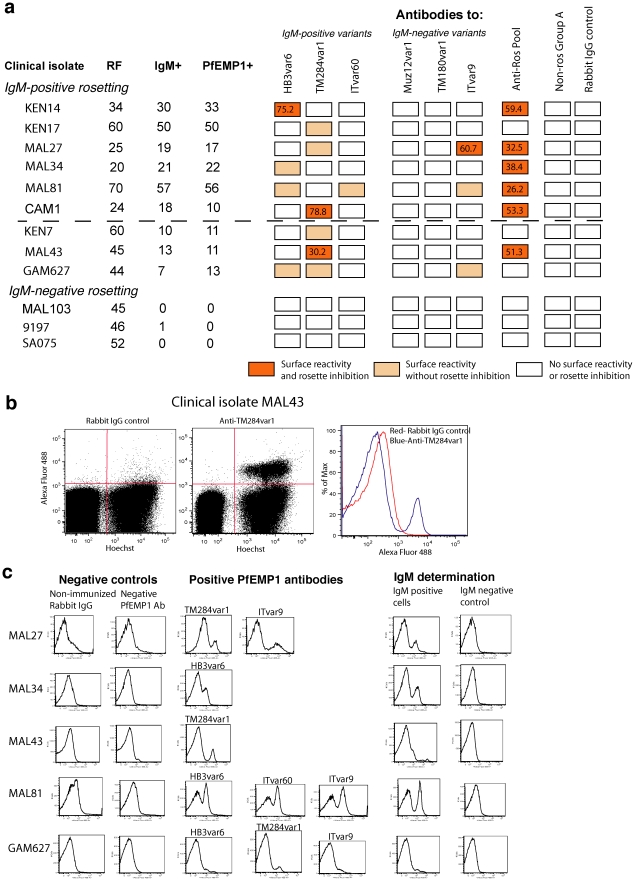
Polyclonal antibodies to PfEMP1 variants from laboratory strains show surface reactivity and rosette inhibition with *P. falciparum* clinical isolates. a) Clinical isolates were tested with PfEMP1 antibodies and controls for surface reactivity by live cell IFA (0.4 mg/ml) and rosette inhibition (1 mg/ml). The rosette frequency (RF), percentage of IgM-positive IEs (IgM+) and percentage of PfEMP1 antibody positive IEs (PfEMP1+, positive with either HB3var6 or TM284var1 antibodies) are shown for each isolate. The PfEMP1 antibodies that showed surface staining with each isolate are indicated by the shaded boxes. Positive surface staining was defined as punctate fluorescence specific to live IEs by IFA (as shown in Figure 2). The percentage rosette inhibition is shown inside each rectangle for all isolate/antibody combinations with >25% rosette inhibition. The controls are as for Figure 4b, and the Anti-Ros Pool is as for Figure 5b. The Anti-Ros pool was tested for rosette inhibition only. The dotted line separates isolates in which RF closely matches the percentage of IgM-positive IEs (above) from those in which the percentage of IgM-positive positive IEs is substantially lower than the rosette frequency (below). b) Flow cytometry of clinical isolate MAL43 with 0.4 mg/ml of total IgG from a non-immunised rabbit (negative control, left panel) and antibodies to TM284var1 (middle panel). IEs stained with Hoechst are in the right half, and antibody-positive IEs stained with Alexa Fluor 488 are in the upper right quadrant. An overlay of histograms (right panel) shows a clear population of stained IEs (blue line, second peak) distinct from the rabbit IgG control (red line, single peak). c) Five clinical isolates were tested by flow cytometry with the PfEMP1 antibody and control panel. The histograms show the negative controls, anti-PfEMP1 positive and IgM-positive IEs. The “negative PfEMP1 Ab” was antibody to TM180var1 and the IgM-negative control was a mouse IgG1 isotype control.

Having shown that polyclonal antibodies to PfEMP1 variants from IgM-positive rosetting parasites show heterologous surface reactivity and biological effector functions against rosetting *P. falciparum* laboratory strains, we carried out a preliminary experiment to examine recognition of clinical isolates from sub-Saharan Africa. The clinical isolates were cryopreserved from previous studies and were selected because they contained at least 20% of IEs in rosettes (see “Materials and Methods” for further details of the clinical isolates origins). Ten clinical isolates were thawed, and all but one contained IgM-positive IEs detected by IFA with an anti-human IgM monoclonal antibody (mAb). For six isolates, the percentage of IgM-positive IEs was very similar to the rosette frequency, suggesting that the majority of rosetting parasites were of the IgM-positive phenotype (Figure 6a, above the dotted line). For three isolates, the percentage of IgM-positive IEs was substantially lower than the rosette frequency, suggesting either a sub-population of IgM-positive rosetting parasites within a larger population of IgM-negative rosetting parasites, or the presence of a sub-population of IgM-positive non rosetting cells (Figure 6a, below the dotted line). One isolate (MAL103) showed no IgM-positive IEs, and two recently culture-adapted, rosette-selected Kenyan isolates (9197 and SA075) were also IgM-negative (Figure 6a).

The panel of PfEMP1 antibodies and controls was tested for surface reactivity with the clinical isolates by IFA (all isolates) and by flow cytometry (five isolates). Positive surface staining was defined as punctate surface fluorescence specific to live IEs in IFA (similar to that shown in Figures 2 and 3) or by a population of Hoechst-positive, Alexa Fluor 488-positive IEs by flow cytometry (Figure 6b). Remarkably, all of the IgM-positive rosetting clinical isolates contained sub-populations of cells that stained with either HB3var6 antibodies or TM284var1 antibodies (Figure 6). The proportions of PfEMP1 antibody positive and IgM-positive cells were closely matched in each isolate (Figure 6a, Pearson correlation r = 0.984, P<0.001). Unfortunately there was insufficient material available to carry out further experiments such as dual colour IFA, therefore we were unable to test directly whether the PfEMP1 antibodies were recognising the IgM-positive IEs. However, the strong positive correlation between the percentages of positive cells, and the similarities in the flow cytometry histograms for IgM-positive and PfEMP1-positive IEs are suggestive that both antibodies are binding to the same sub-population of IEs (Figure 6c).

The clinical isolates were also tested in rosette inhibition assays with the panel of PfEMP1 antibodies and controls. Rosette inhibition was observed in four out of ten isolates, increasing to six isolates when a pool of PfEMP1 antibodies was used (Figure 6a). The IgM-negative clinical isolate (MAL103) and two recently culture-adapted rosette-selected IgM-negative Kenyan strains (9197 and SA075) were not recognized by the PfEMP1 antibodies (Figure 6a). Therefore, in clinical isolates the PfEMP1 antibodies only showed surface reactivity and rosette inhibition of parasites containing populations of IgM-positive IEs.

**Figure 7:**
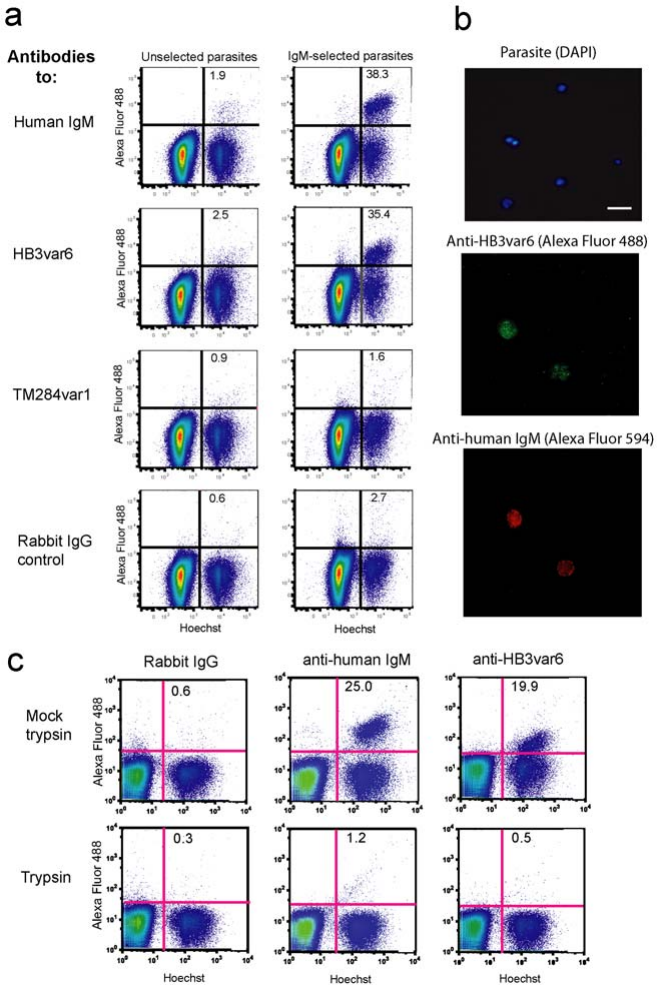
Selection for IgM yields rosetting IEs that are recognised by heterologous polyclonal PfEMP1 antibodies. a) The culture-adapted Kenyan isolate 9197 was selected three times with anti-human IgM coated Dynabeads. Comparison of the unselected and selected lines by flow cytometry showed that the IgM-selected parasites were recognised by cross-reactive PfEMP1 antibodies to HB3var6. The percentage of IEs stained with Alexa Fluor 488 are shown in the upper right quadrant. b) An IFA with dual staining (AlexaFluor 488 anti-rabbit IgG to detect PfEMP1 antibody and AlexaFluor 594 anti-mouse IgG to detect anti-human IgM) shows that the same subpopulation of IEs bound both IgM and HB3var6 antibodies. IEs were stained with DAPI (1 µg/ml; scale bar 10 µm). c) Trypsin sensitivity of surface antigens recognised by HB3var6 antibodies. Trypsinisation is as described in Figure 2. The percentage of IEs stained with Alexa Fluor 488 are shown in the upper right quadrant.

The presence of IgM-positive rosetting variants in diverse parasite isolates was shown further by taking the two recently culture-adapted Kenyan strains 9197 and SA075 which initially showed IgM-negative rosetting (Figure 6a), and selecting them for IgM-binding using magnetic beads coated with anti-human IgM antibodies. After three rounds of selection of strain 9197, a population of IgM-positive rosetting parasites was obtained, which showed surface reactivity with antibodies to HB3var6 but not with antibodies to TM284var1 (9197 IgM-selected, Figure 7a, right column). Dual colour IFA showed that the same subpopulation of IEs bound both IgM and HB3var6 antibodies (Figure 7b). Furthermore the cross-reactive HB3var6 antibodies recognised a trypsin-sensitive surface molecule on 9197IgM+ IEs consistent with PfEMP1 (Figure 7c). For strain SA075, after three rounds of selection a sub-population of IgM-positive cells was obtained (10% of IEs) that stained with antibodies to TM284var1 (not shown).

**Figure 8:**
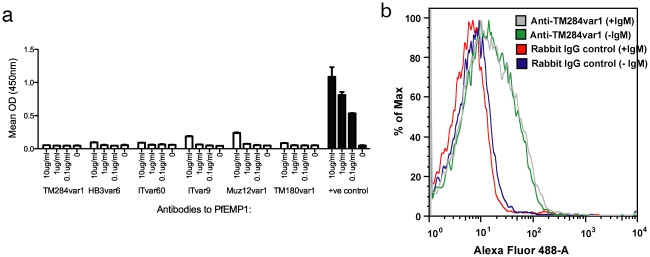
Polyclonal PfEMP1 antibodies do not recognise human IgM. a) ELISA for recognition of human IgM. The positive control is an anti-human IgM antibody. The mean and SD of Optical Density (OD) values from triplicate wells are shown. b) Flow cytometry of IT/PAR+ parasites grown with and without human IgM and stained with TM284var1 antibodies.

We considered the possibility that the strain-transcending effects of the PfEMP1 antibodies against IgM-positive rosetting strains might be explained by the antibodies cross-reacting with human IgM (which is bound to the surface of the IEs from the culture medium). However, the PfEMP1 antibodies did not recognise human IgM in an ELISA (Figure 8a), and the surface reactivity with heterologous parasite strains was maintained when the parasites were grown in the absence of IgM (for example, IT/PAR+ parasites show surface reactivity with TM284var1 antibodies in the absence of IgM as shown in Figure 8b).

## Discussion

In this work the PfEMP1 variants expressed by *P. falciparum* strains representing two major rosetting phenotypes were examined. IgM-positive rosetting parasites were found to express a distinct subset of Group A PfEMP1 variants characterised by a DBLα1.5 or DBLα1.8 N-terminal domain and a triplet of DBLε/DBLζ domains adjacent to the transmembrane region (Figure 1). Polyclonal antibodies raised in rabbits against the N-terminal region of the IgM-positive rosetting variants (HB3var6, TM284var1 and ITvar60) showed surface reactivity against homologous parasites (Figures 2 to [Fig ppat-1002665-g003] 4) and were potent inhibitors of rosetting down to low concentrations (Figure 5). Furthermore, the antibodies had strain-transcending activity at higher concentrations, showing surface reactivity and rosette inhibition against heterologous laboratory strains and clinical isolates sharing the same IgM-positive rosetting adhesion phenotype (Figures 4 to [Fig ppat-1002665-g005] [Fig ppat-1002665-g006] 7). In contrast, IgM-negative rosetting parasites expressed distinct Group A or B/A *var* genes and antibodies raised against them were predominantly variant- and strain-specific, and only rarely recognised heterologous parasite isolates, as shown in previous work [12], [22], [23].

To our knowledge, this is the first report to describe the successful induction of strain-transcending surface-reactive antibodies to PfEMP1 variants implicated in severe childhood malaria. Strain-transcending surface-reactive antibodies against the PfEMP1 variant implicated in malaria in pregnancy (encoded by *var2CSA*) have been described [57], [58], however, *var2CSA* is a unique well-conserved *var* gene with much more limited sequence diversity than that seen in Group A *var* genes [59]. Cross-reactive antibodies to PfEMP1 have also been described using methods such a western blotting [60] and ELISA [12], however, the relationship between recognition of PfEMP1 antigens by these techniques and recognition of native PfEMP1 on the IE surface is not clear. Vigan-Womas *et al* showed that antibodies to three distinct rosetting Group A PfEMP1 variants cross-react by ELISA but do not cross-react in surface reactivity with live cells [12]. Similarly, we found that recognition of DBL domains by ELISA did not correlate well with surface reactivity (Figure S5). This may be due to small amounts of degraded, misfolded or aggregated material within the recombinant protein preparations used in ELISA, or may be due to cryptic epitopes exposed in single recombinant DBL domains that are not exposed in native PfEMP1. These data are important because many researchers use assays based on recombinant proteins to screen for sero-reactivity to PfEMP1. Our data and those of Vigan-Womas *et al* [12] caution against the assumption that results from assays based on recombinant proteins provide information relevant to recognition of native PfEMP1 on the infected cell surface.

The role of strain-transcending antibody responses to PfEMP1 in naturally acquired immunity to malaria remains uncertain. Previous work suggests that African children's’ agglutinating antibody responses to antigens on the IE surface are predominantly variant- and strain-specific [11], [15], [61]. However, other reports suggest that strain-transcending antibodies recognizing conserved epitopes on the surface of IEs can occur in adults exposed to natural infections [15], [62], [63]. Whether the gradual acquisition of immunity to clinical malaria is linked to acquisition of a broad repertoire of antibodies to numerous distinct variant types, or due to development of antibodies to conserved determinants that cross-react against multiple strains remains unresolved. In the case of life-threatening malaria in particular, the role of antibodies to PfEMP1 is unclear. It is known that children become immune to severe malaria after a small number of infections [14], [64], and that severe malaria is associated with the acquisition of antibodies to commonly recognised variants [16], [17], [61]. Current thinking suggests that severe malaria is caused by parasites expressing an antigenically-restricted subset of variant surface antigens [2], probably encoded by Group A *var* genes [29], [30]. Such an “antigenically-restricted” subset of parasites would be expected to have variant surface antigens (probably PfEMP1) showing conserved sequence and/or conserved epitopes that would be recognised by antibodies that show surface reactivity with diverse parasite strains. The findings reported here, that antibodies raised to PfEMP1 variants from IgM-positive rosetting parasites show surface reactivity with diverse parasite strains sharing the same virulence-associated phenotype, may represent the first example of such an “antigenically-restricted” subset of parasites. Our data are suggestive of shared PfEMP1 epitopes amongst the IgM-positive rosetting lab strains and clinical isolates, however, further work will be necessary to identify such epitopes and exclude the possibility that the strain-transcending antibodies are recognising altered host proteins or conserved parasite proteins on the surface of IEs (although no such parasite-derived conserved surface proteins have yet been demonstrated).

All of the parasite lines studied here consisted of heterogeneous mixtures of different variants due to *var* gene switching which occurs spontaneously *in vitro*. This heterogeneous mixture can lead to some difficulties in interpretation of data. For laboratory strains selected for rosetting, the percentage of homologous antibody positive cells varied between 30–75% and closely matched the rosette frequency of the culture. For the IgM-positive rosetting laboratory strains we were able to show by dual staining that the PfEMP1 antibodies (homologous and heterologous) were binding to the IgM-positive IE population (Figure 3 and S3). Ideally future work should focus on parasite strains that have been selected by FACS-sorting and panning with specific antibodies to be essentially mono-variant (>90% single variant) as described by Vigan-Womas *et al* [12]. However, this is technically extremely demanding, especially with parasites expressing Group A-mediated PfEMP1 phenotypes such as rosetting, which are rapidly lost during *in vitro* culture due to switching away from Group A *var* genes [65]. For the clinical isolates, interpretation of data from heterogeneous mixtures of variants is also a problem, and ideally dual staining experiment should be performed to identify unequivocally the subpopulations recognised by homologous and heterologous antibodies. This was not done here, and lack of material prevented further experiments being carried out. However, a strong positive correlation between the percentage of IEs positive for IgM and PfEMP1 antibodies supports the suggestion that the IgM-positive cells were being recognised by the PfEMP1 antibodies, although further work will be needed to test this directly. In addition, further examination of the effector functions of the heterologous PfEMP1 antibodies on clinical isolates would be desirable, including rosette inhibition, phagocytosis and other potential immune clearance mechanisms such as complement mediated lysis. It is not known which of these effector functions would be required for parasite clearance *in vivo*, although it seems likely that surface reactive antibody could lead to clearance via a variety of different mechanisms.

The ability to induce strain-transcending antibodies by immunization with a small number of PfEMP1 NTS-DBLα recombinant proteins as shown here, raises the possibility of developing therapeutic interventions to prevent rosetting. Rosetting is known to be a major *P. falciparum* virulence factor, supported by disease-association studies, animal models and human genetics (reviewed in [19]). However, the exact contribution of rosetting to severe malaria is hard to quantify, and it is unclear how many severe malaria cases could be prevented or treated by an effective anti-rosetting therapy. Other parasite adhesion phenotypes such as platelet-mediated clumping [66], [67] or ICAM-1 binding [68] may contribute to the pathogenesis of severe malaria, although this remains controversial [69]–[72]. A complete understanding of the patho-physiological mechanisms leading to severe malaria and the role of specific adhesion phenotypes in these pathways remains elusive, and is an important area for further research. Currently, rosetting is the most well-substantiated virulence factor in human malaria, and human genetic studies showing that rosette-reducing erythrocyte polymorphisms reduce the odds ratio for severe malaria by up to two-thirds [38], [39], suggest that there is considerable clinical benefit to reducing rosetting. The strain-transcending antibodies against IgM-positive rosetting parasites reported here were generated by immunizing rabbits with NTS-DBLα domains of PfEMP1. If similar responses could be raised in humans, this would raise the possibility of an anti-rosetting vaccine to prevent some cases of severe malaria. Alternatively, if shared PfEMP1 epitopes can be identified and mapped, it may be possible to target them with small molecule drugs to disrupt rosettes, and so develop an adjunctive therapy for severe malaria. It is interesting to note that because of the effect of ABO blood group on rosetting (rosettes form poorly in group O erythrocytes [33], [73] and group O individuals are partially protected from severe malaria [39], [74]), any anti-rosetting intervention would be predicted to have most pronounced clinical benefit for patients with non-O blood groups. Group O individuals can still suffer from severe malaria however, therefore although anti-rosetting interventions clearly have potential for prevention or adjunctive therapy of severe disease [19], they are likely to be most useful as part of a cocktail of anti-severe disease measures.

Further development of anti-rosetting therapies would be aided by a more detailed understanding of the role of particular rosetting phenotypes in the development of severe malaria. In particular, the relative contributions of IgM-positive and IgM-negative rosetting phenotypes to severe malaria have received little attention to date. The only study to examine IgM-positive rosetting in clinical isolates with specific reagents found a strong positive correlation between IgM-binding and rosetting and severe disease, although rosetting was the more strongly-associated variable [41]. Other studies of rosetting and severe malaria (reviewed in [19]) have not investigated the IgM-binding phenotype of the parasites, therefore more research in this area is desirable.

The biological function of the human IgM bound to the surface of *P. falciparum* IEs has also received relatively little attention to date [75]. Initial studies suggested that rosetting parasites can bind both IgG and IgM from normal human serum and that this is important for strengthening rosettes [40], [76]. However, subsequent studies using specific mAb reagents to detect human immunoglobulins showed only IgM, but not IgG on the surface of rosetting IEs [41]. Non-immune IgM (but not IgG) was also detected on the IE surface of CSA-binding parasites implicated in placental malaria [46], whereas parasite strains showing other common adhesion phenotypes such as CD36-binding, ICAM-binding and platelet-mediated clumping do not bind non-immune immunoglobulins [41]. Further studies of rosetting and CSA-binding parasites confirmed that non-immune IgG does not bind to IEs, and used domain swap antibodies based on an IgG backbone to show that the Cμ4 domain of IgM is required for binding to PfEMP1 [50]. Recent data from parasites expressing *var2CSA* suggest that IgM-binding might be an immune evasion mechanism that makes PfEMP1 less accessible to specific antibodies [44].

One unexplained feature of the current data is why antibodies to IgM-positive rosetting PfEMP1 variants show strain-transcendent activity, whereas antibodies to IgM-negative rosetting PfEMP1 variants do not, despite apparently equivalent amino acid diversity in the two sets of variants. We considered the possibility that the IgM itself could be the cause of the cross-reactivity, however we showed that the PfEMP1 antibodies did not recognise human IgM in an ELISA, and the PfEMP1 antibodies still recognize heterologous strains when the parasites were grown in the absence of human IgM (Figure 8). It may be that a small sequence motif such as one of the homology blocks described by Rask *et al* [6] present only in the IgM-positive variants may explain the cross-reactivity. Additional examples of IgM-positive rosetting variants and detailed mapping of epitopes recognised by strain-transcending antibodies will be needed to investigate this possibility. Alternatively, it is possible that the binding of IgM to PfEMP1 affects its tertiary or quaternary structure, making it more accessible to antibodies directed against the N-terminus of the molecule.

Another poorly understood aspect of rosetting is the precise contribution of different parts of the PfEMP1 molecule to rosette formation, and the relationship between the IgM-binding and erythrocyte-binding regions of PfEMP1. Previous data show that the primary receptor-ligand interaction in rosetting occurs between NTS-DBLα of specific PfEMP1 variants and receptors on uninfected Es [12], [22], [23]. However, the IgM-binding region of PfEMP1 maps to a different part of the molecule (the final or penultimate DBLε or DBLζ domain before the transmembrane region [49], [50] and AG and JAR, unpublished data). IgM is thought to enhance rosetting by strengthening the adhesive interactions between infected and uninfected Es [40], [42], [43]. Whether it does this by “bridging” between the IE and receptors on uninfected Es [43], or by altering the conformation of PfEMP1 to enhance its affinity for erythrocyte receptors is unclear. However, IgM on its own is not sufficient to cause rosetting; for example, CSA-binding parasites bind IgM but do not rosette [46]. Based on our current data, we suggest that antibodies to NTS-DBLα block rosetting by directly interfering with the receptor-ligand interaction between PfEMP1 and erythrocyte receptors. The NTS-DBLα antibodies do not affect IgM binding, because dual-staining experiments showed that human IgM is detected on the surface of rosetting IEs even in the presence of PfEMP1 antibodies (Figure 3 and S3). Exactly how IgM-binding influences PfEMP1 function and contributes to rosette formation is not clear and will require further work.

One of the main findings from this study is the identification of a clear subset of Group A PfEMP1 variants expressed by IgM-positive rosetting parasites, exemplified by variant HB3var6 from strain HB3R+, variant TM284var1 from strain TM284R+ and variant ITvar60 from strain IT/PAR+. ITvar60 has previously been linked to rosetting in two other IT/FCR3-derived parasite lines [77], [78], and is confirmed here as an IgM-positive rosetting variant. This subset of Group A PfEMP1 variants from IgM-positive rosetting parasites show two out of eight possible subclasses of DBLα1 domain (DBLα1.5 or DBLα1.8) [6] and a set of three DBLε/DBLζ domains adjacent to the transmembrane region (Figure 1). Rask et al [6] recently presented an alternative way of assessing PfEMP1 types by looking at “domain cassettes” (sets of PfEMP1 domains that usually occur together). They identified seven domain cassettes commonly found in Group A *var* genes [6]. Our data suggest that two of these domain cassettes are linked to the IgM-positive rosetting phenotype: domain cassette 16, characterised by DBLα1.5 linked to CIDRδ delta as seen in HB3var6, and domain cassette 11 characterised by DBLα1.8 linked to CIDRβ2 and DBLγ7 as seen in ITvar60 and TM284var1. The clinical isolates we studied showed surface reactivity with either HB3var6 antibodies (DBLα1.5/domain cassette 16) or TM284var1 antibodies (DBLα1.8/domain cassette 11), but rarely with both (Figure 6). These data are suggestive that these two main DBLα1 types may underlie the IgM-positive rosetting phenotype in diverse field isolates, although further sequence information is needed to substantiate this idea.

Other variants with similar PfEMP1 architecture to the IgM-positive rosetting variants described here can be seen in the genome of a recently sequenced *P. falciparum* strain IGH (*IGHvar12*, *IGHvar 22* and *IGHvar 24* [6]). Furthermore, an ITvar60-like variant occurs in the sequenced *P. falciparum* strain D10 from Papua New Guinea (http://www.broadinstitute.org). Taken together, these data suggest that variants with the IgM-positive rosetting type of PfEMP1 architecture occur commonly in geographically diverse *P. falciparum* isolates. One limitation of the current study was that there was insufficient material from the clinical isolates to allow us to identify and sequence their expressed *var* genes. The selection of IgM-positive rosetting parasites from culture-adapted clinical isolates (Figure 7) will allow us to examine their *var* genes in further detail. The correct identification of rosette-specific variants (Table S1) and sequencing of full-length *var* genes remains a laborious and time-consuming process for isolates that do not have a full genome sequence available. However, wider studies of PfEMP1 architecture and sequence from rosetting clinical isolates will be essential for a full understanding of how the antibody cross-reactivity documented here relates to sequence diversity and PfEMP1 type.

In summary, these data show that antibodies raised against a subset of Group A PfEMP1 variants from IgM-positive rosetting laboratory strains show surface reactivity and rosette inhibition against heterologous parasites sharing the same adhesion phenotype. These data suggest shared surface epitopes amongst *P. falciparum* isolates with a shared virulence-associated phenotype; a phenomenon that may underlie the epidemiological observations that children acquire immunity to life-threatening malaria after a small number of infections [14], [64]. Most importantly, the ability to elicit strain-transcendent antibodies by immunizing with key PfEMP1 variants underlying a virulence phenotype, suggests that designing interventions to prevent severe malaria is a realistic goal.

## Materials and Methods

### Ethics statement

Collection of clinical isolates (blood samples) from malaria patients was carried out in accordance with the Declaration of Helsinki. Written informed consent was obtained from the patients' parents or guardians and was approved by the Lothian Regional Ethical Review Committee (LREC//2002/4/34), the KEMRI Ethical Review Committee, the Gambia Government/MRC Laboratories Joint Ethics Committee, the Cameroon Ministry of Public Health Regional Ethics committee and the University of Bamako Institutional Review Board. Animal immunisations were carried out commercially by BioGenes GmbH (Berlin, Germany) according to European Union guidelines 86/609/EWG of 24.11.1986 and the European Agreement of 18.3.1996 for protection of animals used for scientific purposes.

### Parasites and parasite culture

The *P. falciparum* laboratory strains (HB3, TM284, IT/PAR+, Muz12, IT/R29 and TM180) were cultured in supplemented RPMI with 10% pooled normal human serum as described [79]. Each strain was separated into isogenic rosetting (R+) and non-rosetting (R−) sub-populations by gelatin flotation or centrifugation though 60% Percoll [48]. For consistency, the rosette-selected strains are here designated “strain name R+” throughout (eg. HB3R+) except for IT/R29 (where the “R” indicates rosetting). Repeated rosette selection [48] of the R+ strains (2–3x per week) was required to maintain the rosetting phenotype, which is otherwise rapidly lost *in vitro*. The rosette frequency is the percentage of IEs in rosettes out of 200 IEs assessed by microscopy of an ethidium-bromide-stained wet preparation as described [80]. The rosette frequency of selected parasites varied between 30–75% depending on the frequency of rosette selection and *var* gene switching (which occurs spontaneously *in vitro*). The IgM-binding phenotype of the rosetting strains was determined by immunofluorescence assay (IFA) with an anti-human IgM mAb (Serotec MCA1662 1/500 dilution) as described [41]. The IgM phenotype of TM284R+ and IT/PAR+ (IgM-positive rosetting) and IT/R29 and TM180R+ (IgM-negative rosetting) has been reported previously [41]. HB3R+ shows IgM-positive rosetting (Figure 3 and Table S9) whereas Muz12R+ shows predominantly IgM-negative rosetting (Table S9). With some strains (eg. TM284R+ and HB3R+) the IgM-positive IEs can be seen to be in rosettes after the IFA. However, in others (eg. IT/PAR+) the rosettes are disrupted by repeated washing during the IFA, and in these cases the designation of IgM-positive rosetting relies upon consistent strong positive correlation between the percentage of rosette-forming and IgM-positive IEs in repeated experiments. All cultures were checked regularly to exclude mycoplasma contamination [81]. The parasites were genotyped with primers to MSP1, MSP-2 and GLURP [82] and were genetically distinct apart from IT/PAR+ and IT/R29 which share the same genotype but transcribe different predominant PfEMP1 variants.

Other parasite strains used were unselected HB3 and 3D7 (CD36-binding), IT/A4 (CD36 and ICAM-1 binding) and three strains selected for binding to human brain endothelial cells (HB3-HBEC, 3D7-HBEC and IT-HBEC [83]). These strains all have <5% IgM-positive IEs by IFA.

Clinical isolates were from Cameroon (CAM1), Kenya (KEN7, KEN14, KEN17, 9197, SA075), Mali (MAL27, MAL34, MAL43, MAL81, MAL103) and The Gambia (GAM627). All clinical isolates were cryopreserved from previous studies and were selected because records showed they had a rosette frequency of 20% or higher in the first asexual cycle *in vitro* when fresh. The Malian isolates were collected in Bamako in 1996 as part of a pilot study on rosetting and malaria severity in Mali. Kenyan isolates KEN7, KEN14 and KEN17 were collected as part of a case-control study on severe malaria [84], while 9197 and SA075 were from studies on *var* gene diversity in Kenya [85]. The Gambian isolate GAM627 was collected in 2009–2010 as part of a study on rosette-inhibiting drugs (Rowe *et al*, unpublished data). During the Gambian study, 23 isolates from severe malaria patients were collected of which seven had >20% rosette frequency and >1% parasitaemia, but only one of these was cryopreserved (GAM627) and therefore suitable for use in this study. The Cameroonian isolate CAM1 was collected in 2009–10 as part of a study on *var* gene transcriptional profiling and clinical malaria severity (Rowe *et al*, unpublished data). Of 38 isolates collected from severe and uncomplicated malaria patients, only three showed >20% rosette frequency >1% parasitaemia and only one of these (CAM1) grew after thawing.

For all clinical isolates, an aliquot was put into culture at the time of original collection and its rosette frequency determined as described [33]. The remainder of the sample was cryopreserved within 12 hours of the blood sample being drawn and was not cultured prior to freezing. These cryopreserved samples were used for this study. The isolates were thawed as described [80] and were tested for surface reactivity and rosette inhibition with PfEMP1 antibodies and controls as described for laboratory strains. Experiments were carried out in the first cycle after thawing, except for 9197 and SA075 which had been adapted to culture, cloned and selected for rosetting over 3–4 months of *in vitro* growth. The IgM-binding phenotype of the rosetting clinical isolates was not determined during their initial collection in the studies outlined above, but was determined after thawing by IFA with an anti-human IgM mAb as described above for the laboratory strains.

### *Var* gene expression profiling and *var* gene sequencing

RNA extraction and *var* gene expression profiling were carried out as described previously [27] and in Table S1. The full-length sequence of each predominant rosette-specific *var* gene was derived from the sequence tag by: a) extraction from parasite genome databases (HB3 at http://www.broadinstitute.org and IT at www.sanger.ac.uk) b) PCR-walking, cloning and sequencing using degenerate primers to upstream and downstream PfEMP1 regions [86] for *Muz12var1*. c) PCR-walking, cloning and sequencing using vectorette libraries [22] for *TM284var1* and *TM180var1*. The GenBank Accession numbers for the sequences studied here are Y13402 (*ITvar9/R29var1*), EF158099 (*ITvar60*), JQ684046 (*TM284var1*), JQ684047 (*TM180var1*) and JQ684048 (*Muz12var1*). The *HB3var6* sequence can be obtained from http://www.broadinstitute.org/annotation/genome/plasmodium_falciparum_spp/MultiHome.html gene reference PFHG_02274.1. DNA sequence analysis was done using DNAstar Lasergene (DNAstar Inc.)

### Northern blotting

RNA extraction and Northern blotting of isogenic rosetting and non-rosetting pairs of parasites was carried out with Digoxigenin-labelled RNA probes as described [50]. RNA (1.5 µg per lane) was electrophoresed on a 1.2% agarose/1.1% formaldehyde gel and transferred onto a nitrocellulose membrane. For each parasite strain, the blot was hybridised with a specific RNA probe representing one DBL domain from the homologous rosette-specific *var* gene, as well as an exon II probe to detect all *var* genes. Probes were generated with the following primers: *HB3var6*, CIDRδ, forward 5′-*tctcgtcagctg*gatgaaagtaattctcatag-3′ (the italicized region indicates a restriction site added to the primers for other experiments; the gene specific sequence is in regular font), reverse 5′-*acgagtgggccc*tccaataagtttcttcaccat-3′; ITvar60, 5^th^ DBL domain, forward 5′-*tctcgtcagctg*gaggaatatcctgaagaatac-3′, reverse 5′-*acgagtgggccc*caaattacattcaccttc-3′; *Muz12var1*, DBLγ, forward 5′-gtagcagaagatggtgcttg-3′, reverse 5′-ctttccactttataagcc-3′; *TM180var1*, DBLβ, forward 5′-gaacagggtgaaaacacta-3′, reverse, 5′-caagcttgtgtgcacctctg-3′; Exon II, forward 5′- aaaaaaccaaagcatctgttggaaatttat-3′, reverse 5′-gtgttgtttcgactaggtagtaccac-3′. High stringency conditions (specific *var* gene probes) were hybridisation at 58°C overnight, followed by washing at 62°C with 0.5× SSC/0.1%SDS for 45 mins followed by 0.25× SSC/0.1%SDS for 45 mins. Moderate stringency conditions (Exon II probe) were hybridisation at 52°C overnight, followed by washing at 55°C with 0.5× SSC/0.1%SDS for 45 mins followed by 0.25× SSC/0.1%SDS for 45 mins.

### Recombinant proteins and polylconal antibodies

Recombinant proteins were produced as described previously [13]. The domain boundaries for the NTS-DBLα recombinant proteins for each rosette-specific variant were as follows: HB3var6 Met1-Pro473; TM284var1 Met1-Pro457; ITvar60 Met1-Pro464; Muz12var1 Met1-Pro458; TM180var1 Met1-Pro485. The non-rosetting Group A PfEMP1 variant HB3var3 (Met1-Pro468) was used as a control (Claessens and Rowe *et al*, submitted). The His-tags used for protein purification were cleaved by TEV protease before immunization as described [13]. Each protein was used to immunize two rabbits which had been pre-screened as described [13] to avoid animals with pre-existing natural antibodies to human erythrocytes or malaria parasites. Immunization and serum collection were carried out by BioGenes GmBH (Berlin, Germany). Rabbits were immunized with 250 µg of protein on day 0 and with 100 µg on day 7, 14 and 28 and 49. Immunizations were carried out using an adjuvant developed by Biogenes GmbH that contained 0.23% of lipopolysaccharides of the blue-green algae *Phormidium* spp, 92.8% mineral oil, 3.48% Tween-20, 3.48% Span-80. Final bleed antisera were collected on day 56. Total IgG purification was carried out by Biogenes, and all antibody concentrations given in µg/ml throughout this manuscript are concentrations of total IgG.

### Immunofluorescence assays (IFA)

Immune and pre-immune sera were tested in IFA with live IEs as described [13], [50]. Out of each pair of immunized rabbits, the serum giving the brightest fluorescent signal with the lowest background was chosen for purification of total IgG. In all cases, both rabbit sera gave positive PfEMP1-staining, with only minor differences in intensity of staining. The percentage of IEs staining with the PfEMP1 antibodies and the anti-human IgM mAb was assessed by counting 100 DAPI-stained IEs per slide. IFA slides were viewed using a Leica DM LB2 fluorescence microscope and images taken with a Leica DFC300FX digital camera. Images were handled using Adobe Photoshop and underwent cropping and minor adjustments to brightness and contrast. All adjustments were applied equally to PfEMP1 antibody and control images.

### Flow cytometry

Staining for flow cytometry was carried out as for IFA [13], [50], except that 1.25 µg/ml Hoechst 33342 stain (Sigma) was used instead of DAPI to stain IEs and 50 µg/ml fucoidan was added after the secondary incubation washes to disrupt rosettes. Staining and washes were carried out on live (unfixed) cells, but before FACS analysis, cells were fixed with 0.5% paraformaldehyde, with 50 µg/ml fucoidan added to prevent rosettes from re-forming. 500,000 events per sample were analyzed on a Becton-Dickinson LSRII flow cytometer. Flow cytometry data were analyzed using FlowJo software (Tree Star Inc.).

### Flow cytometry of trypsinised parasite cultures

Parasite cultures of mature pigmented trophozoites with a rosette frequency of at least 30% were used for trypsinisation experiments. 20 µl of packed cells from a parasite culture were centrifuged and washed twice in incomplete RPMI. The cells were resuspended in 500 µl of 10 µg/ml of TPCK-trypsin (Sigma) or incomplete RPMI (called “mock trypsin”), mixed and incubated at room temperature for 5 mins. The reaction was stopped by adding 500 µl of 1 mg/ml of Soybean trypsin inhibitor (Sigma) to the trypsin-treated and mock trypsin samples, which were mixed and incubated at room temperature for 5 mins. The samples were centrifuged at 4000 rpm for 2 mins and washed twice in incomplete RPMI and once in PBS. The cells were resuspended in PBS containing 1% BSA and 1.25 µg/ml of Hoechst, and staining was carried out as described for IFA and flow cytometry above. All antibodies were used at a final concentration of 100 µg/ml except for anti-NTS-DBLα (HB3var6), which was used at 400 µg/ml when tested against the parasite strain 9197.

### Dual colour IFA

Dual colour IFA were carried out to test whether the homologous and heterologous (cross-reactive) antibodies bind to the IgM-positive rosetting IE population. Staining was carried out as above with the primary incubation containing both 1/500 of mouse monoclonal anti-human IgM (Serotec MCA 1662) and 20 µg/ml of rabbit polyclonal NTS-DBLα antibodies. Secondary incubations were carried out with a mixture of 1/1000 dilution of highly cross-absorbed Alexa Fluor 488 goat-anti rabbit IgG (Invitrogen) and 1/1000 dilution of highly cross-absorbed Alexa Fluor 594 goat anti-mouse IgG (Invitrogen). In addition to a secondary only control, and a mouse isotype control plus rabbit IgG control, combinations of single stains were used to rule out any non-specific binding of Alexa Fluor 488 anti-rabbit to mouse anti-human IgM and of Alexa Fluor 594 anti-mouse to rabbit IgG. The percentage of PfEMP1-positive cells that were positive for IgM and vice versa were determined by counting 100 positive IEs per slide.

### Rosette inhibition experiments

*P. falciparum* cultures at ring stage were incubated overnight with antibodies and controls at various dilutions, and rosetting assessed the next day by microscopy as described [13]. Antibodies at the highest concentration (1 mg/ml) were dialysed before use to remove non-specific growth-inhibitory factors. Approximately 200 µl of total IgG was added to a dialysis cassette (Pierce) and dialysis was carried out against 500 ml of PBS overnight at 4°C. The rosette frequency (RF) is the percentage of mature (pigmented trophozoite)-IEs binding two or more uninfected Es from 200 IEs counted.

### Phagocytosis assays

Phagocytosis experiments with Thp-1 cells were as described previously [13] except that fucoidan (200 µg/ml) was used for parasite purification and rosette disruption. The positive control was parasite culture opsonized with 90 µg/ml of a rabbit anti-human erythrocyte antibody (ABCAM ab34858). Muz12var1 antibodies were not included in the phagocytosis assays because they show some background binding to uninfected Es.

### Selection for IgM-positive IEs

Parasites were selected for IgM-positive IEs using M-450 Epoxy Dynabeads (Dynal) coated with a mouse anti-human IgM mAb (Serotec MCA1662) as described [87].

### ELISA

The ability of PfEMP1 antibodies to cross react with human IgM was tested using purified human IgM (5 µg/ml, Rockland) coated onto an ELISA plate at 4°C overnight. After blocking for 1 hour in PBS containing 0.05% Tween 20 (PBST) and 5% milk, wells were incubated with 10, 1 and 0.1 µg/ml of rabbit polyclonal NTS-DBLα antibodies in PBST containing 1% milk (PBSTM). After 1 hour incubation at room temperature, wells were washed with PBST and incubated with 1∶10,000 anti-rabbit IgG-HRP (Sigma) in PBSTM for a further hour. After washing as above, reactions were developed by incubating the wells with substrate 3,3′,5,5′-tetramethylbenzidinedihydrochloride (Sigma) according to the manufacturer's instructions and absorbance was measured at a wavelength of 450 nm. As a positive control, a rabbit anti-human IgM F(ab')2-HRP (DAKO) was used at 1∶100 (10 αg/ml), 1∶1000 (1 µg/ml) and 1∶10000 (0.1 µg/ml). A set of ELISA experiments were carried out to test the ability of rabbit polyclonal NTS-DBLα antibodies to recognise homologous and heterologous recombinant NTS-DBLαproteins. The method was as described for the IgM ELISA except that wells were coated with 2 µg/ml recombinant NTS-DBLαprotein and antibodies were used at a range of concentrations from 0–10 µg/ml. Blocking, washing, incubation and detection was carried as described for the IgM ELISA.

### Heterologous surface reactivity of PfEMP1 antibodies in the absence of IgM

Pooled human serum was depleted of IgM by three successive rounds of incubation for 45 mins at room temperature on a rotating wheel (15 rpm) with an equal volume of anti-human IgM (μ-chain specific)-agarose (Sigma A9935). The absence of IgM in the absorbed serum was confirmed by western blotting with an anti-human IgM monoclonal antibody. IT/PAR+ parasites were grown from ring stage overnight in supplemented RPMI with 10% IgM-depleted serum, and an aliquot (positive control culture) was incubated with 1 mg/ml of human IgM (Calbiochem) for 1 hour at 37°C. The IgM-negative and IgM-positive cultures were then washed and testing for surface reactivity with heterologous PfEMP1 antibodies to TM284var1 NTS-DBLα by flow cytometry as described above.

### Software

Graphing and statistical analysis were done using Prism (GraphPad Software).

## Supporting Information

Figure S1 **Alignment of NTS-DBLα domains from rosetting PfEMP1 variants.** Sequences were aligned by Clustal W. Amino acid residues that match the consensus sequence are shaded black. The rosetting variants are as described in this work plus ITvar9 [22], Palo Alto Var O [23] and PF13_0003 [12].
(TIF)

Figure S2 **Western blotting with polyclonal antibodies to PfEMP1.** Triton-X-100 souluble (Tx) and Triton-X-100 insoluble/SDS soluble (SDS) extracts of parasite cultures and uninfected Es (RBC) were electrophoresed on 3–8% Tris-acetate gels, transferred to PVDF membrane and probed with antibodies to PfEMP1. a) 6H1 PfEMP1 mAb (1/1000) tested on IT/PAR+ parasites. b) 6H1 PfEMP1 mAb (1/1000) tested on IT/R29 parasites. c) 6H1 PfEMP1 mAb (1/1000) tested on IT/PAR+ parasites. d) ITvar60 NTS-DBLα rabbit polylconal antibodies (1/15,000) tested on IT/PAR+ parasites. Parasite-specific high molecular weight bands consistent with PfEMP1 are arrowed. See Text S1 for further details and methods.
(TIF)

Figure S3 **Homologous and heterologous polyclonal antibodies to PfEMP1 recognize IgM-positive IEs.** a) Parasite strain TM284R+ was stained in a live cell IFA with a mixture of rabbit polylconal antibodies to PfEMP1 (homologous or heterologous) at 20 µg/ml and mouse anti-human IgM mAb (Serotec MCA1662 1/500 dilution). Secondary incubation was with a mixture of Alexa Fluor 488 conjugated anti-rabbit IgG (1/1000) and Alexa Fluor 594-conjugated anti-mouse IgG (1/1000). IEs were stained with DAPI (1 µg/ml; scale bar 10 µm). IgM-positive IEs (right column) show punctate/rim surface fluorescence with both homologous antibodies (anti-TM284var1, 2nd row, middle column) and heterologous antibodies (anti-ITvar60, 3rd row, middle column). 94–100% of the PfEMP1 antibody-positive cells were IgM-positive, and 100% of the IgM-positive cells were PfEMP1-antibody positive. At this concentration TM180var1 antibodies give very faint punctate fluorescence on IgM-positive IEs (5th row, middle column) whereas HB3var6 antibodies are negative (4th row, middle column). At higher concentrations (100–400 µg/ml) both TM180var1 and HB3var6 antibodies stain IgM-positive IEs. b) Specificity controls (with parasite strain TM284R+ as above) show that the Alexa 488 conjugated anti-rabbit IgG secondary does not recognise the mouse anti-human IgM mAb (top row) and that the Alexa 594-conjugated anti-mouse IgG does not recognise the rabbit polyclonal antibodies (bottom row). Camera exposure settings and image handling were identical for PfEMP1 antibodies and controls.
(PDF)

Figure S4 **Trypsin-sensitivity of IE surface molecules recognized by heterologous polyclonal PfEMP1 antibodies.** a) Flow cytometry of live IEs of *P. falciparum* strain TM284R+ stained with homologous (anti-TM284var1) and heterologous (anti-ITvar60 and anti-TM180var1) PfEMP1 antibodies (100 µg/ml of total IgG). The negative control was IgG from a non-immunized rabbit (rabbit IgG). IEs were stained with Hoechst and rabbit IgG bound to the surface of IEs was detected with highly cross-absorbed Alex Fluor 488-conjugated anti-rabbit IgG at 1/500 dilution. The IE molecules recognised by PfEMP1 antibodies were sensitive to trypsin (right column) (10 µg/ml trypsin for 5 mins at room temperature (RT), followed by 1 mg/ml of trypsin inhibitor for 5 mins at RT). b) Flow cytometry of live IEs of *P. falciparum* strain IT/PAR+ stained with homologous (anti-ITvar60) and heterologous (anti-TM284var1 and anti-HB3var6) PfEMP1 antibodies (100 µg/ml of total IgG). Rabbit IgG control and dilutions as above. Trypsin treatment was as above except using 1 mg/ml trypsin. For IT/PAR+ the IE molecules recognised by both homologous and heterologous PfEMP1 antibodies are trypsin-resistant.
(PDF)

Figure S5 **Recognition of recombinant NTS-DBLα domains by homologous and heterologous antibodies by ELISA.** Recombinant NTS-DBLα domains of the PfEMP1 variants encoded by *HB3var6*, *TM284var1*, *ITvar60*, *Muz12var1*, *TM180var1* and *ITvar9* were coated at 2 µg/ml and incubated with NTS-DBLα antibodies over a range of concentrations from 0–10 µg/ml. Binding was detected using 1∶10,000 dilution of anti-rabbit IgG-HRP (Sigma). The mean and SD of Optical Density (OD) values from triplicate wells are shown. a) rabbit IgG control, b) anti-HB3var3, c) anti-ITvar9, d) anti-TM180var1, e) anti-Muz12var1, f) anti-TM284var1, g) anti-HB3var6 and h) anti-ITvar60.
(PDF)

Figure S6 **Opsonisation and induction of phagocytosis by polyclonal PfEMP1 antibodies.** IEs were stained with ethidium bromide and opsonised with PfEMP1 antibodies over a range of concentrations (1.56–400 µg/ml) before incubation with the monocytic cell line Thp-1. Thp-1 cells that phagocytosed IEs were detected by flow cytometry. a) parasite strain TM284R+, b) parasite strain HB3R+, c) parasite strain IT/R29, d) parasite strain TM180R+. Data are shown as percentage of the positive control opsonised with a rabbit anti-human erythrocyte antibody (ABCAM ab34858 at 90 µg/ml). The “Non Ros Group A” negative control consists of antibodies to HB3var3, a PfEMP1 variant that is not involved in rosetting. HB3R+ parasite culture contains a subpopulation of non-rosetting IEs expressing HB3var3 (see Table S1) which explains why phagocytosis was induced in this case. The “Control Rabbit IgG” is a negative control consisting of IgG from a non-immunized rabbit.
(PDF)

Table S1 **Identification of a predominant rosette-specific** ***var*** **gene by transcriptional profiling of isogenic rosetting (R+) and non-rosetting (R−) HB3 parasites.**
(DOC)

Table S2 **Pair-wise amino acid identities for NTS-DBLα, CIDR1 and DBLγ from rosetting PfEMP1 variants.**
(DOC)

Table S3 **Pair-wise amino acid identities for DBLε from rosetting PfEMP1 variants.**
(DOC)

Table S4 **Pair-wise amino acid identities for DBLζ from rosetting PfEMP1 variants.**
(DOC)

Table S5 **Pair-wise amino acid identities for DBLβ from rosetting PfEMP1 variants.**
(DOC)

Table S6 **Pair-wise amino acid identities for DBLδ from rosetting PfEMP1 variants.**
(DOC)

Table S7 **Pair-wise amino acid identities for CIDR2 from rosetting PfEMP1 variants.**
(DOC)

Table S8 **Summary of PfEMP1 antibody activity against homologous parasite strains.**
(DOC)

Table S9 **IgM phenotype of HB3R+ and Muz12R+ parasites.**
(DOC)

Text S1 **Western blots to detect ITvar60 PfEMP1.**
(DOC)

Text S2 **Reactivity of PfEMP1 antibodies with recombinant NTS-DBLα recombinant proteins by ELISA.**
(DOC)
