# Supplementary figures and images for: Induction of Strain-Transcending Antibodies Against Group A PfEMP1 Surface Antigens from Virulent Malaria Parasites
Source: PLoS Pathog. 2012 Apr 19;8(4):e1002665. doi: 10.1371/journal.ppat.1002665 (PMC3330128; doi:10.1371/journal.ppat.1002665)

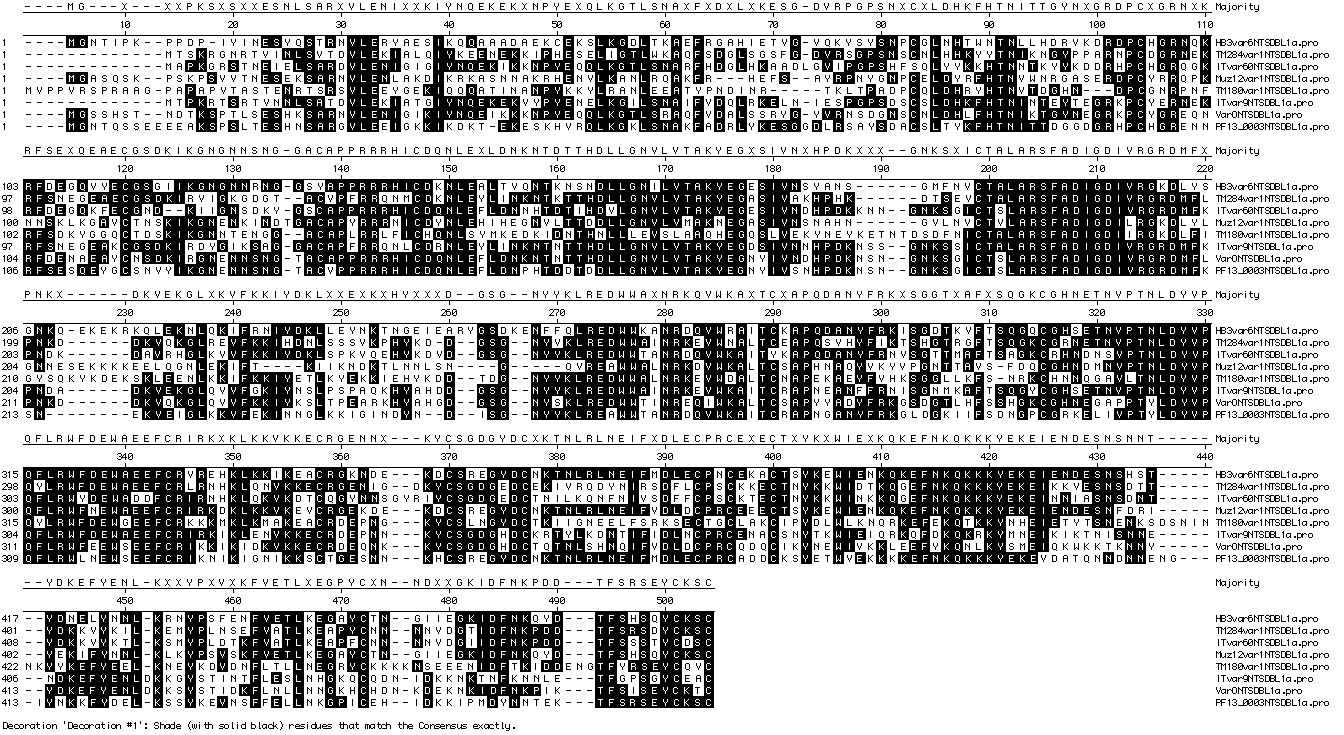

Supplement: Figure S1 — Alignment of NTS-DBLα domains from rosetting PfEMP1 variants. Sequences were aligned by Clustal W. Amino acid residues that match the consensus sequence are shaded black. The rosetting variants are as described in this work plus ITvar9 [22], Palo Alto Var O [23] and PF13_0003 [12]. (TIF) [file ppat.1002665.s001.tif]

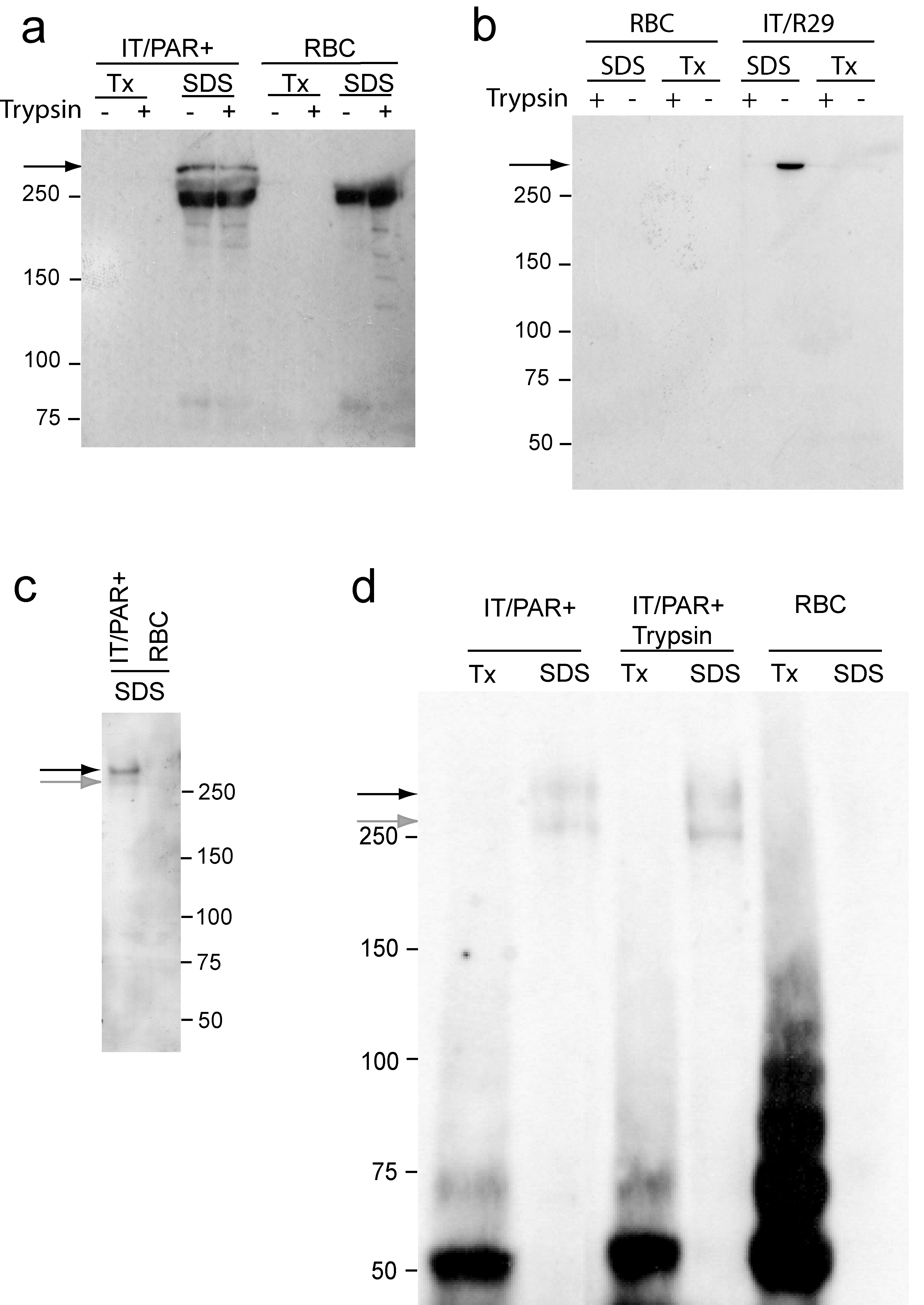

Supplement: Figure S2 — Western blotting with polyclonal antibodies to PfEMP1. Triton-X-100 souluble (Tx) and Triton-X-100 insoluble/SDS soluble (SDS) extracts of parasite cultures and uninfected Es (RBC) were electrophoresed on 3–8% Tris-acetate gels, transferred to PVDF membrane and probed with antibodies to PfEMP1. a) 6H1 PfEMP1 mAb (1/1000) tested on IT/PAR+ parasites. b) 6H1 PfEMP1 mAb (1/1000) tested on IT/R29 parasites. c) 6H1 PfEMP1 mAb (1/1000) tested on IT/PAR+ parasites. d) ITvar60 NTS-DBLα rabbit polylconal antibodies (1/15,000) tested on IT/PAR+ parasites. Parasite-specific high molecular weight bands consistent with PfEMP1 are arrowed. See Text S1 for further details and methods. (TIF) [file ppat.1002665.s002.tif]

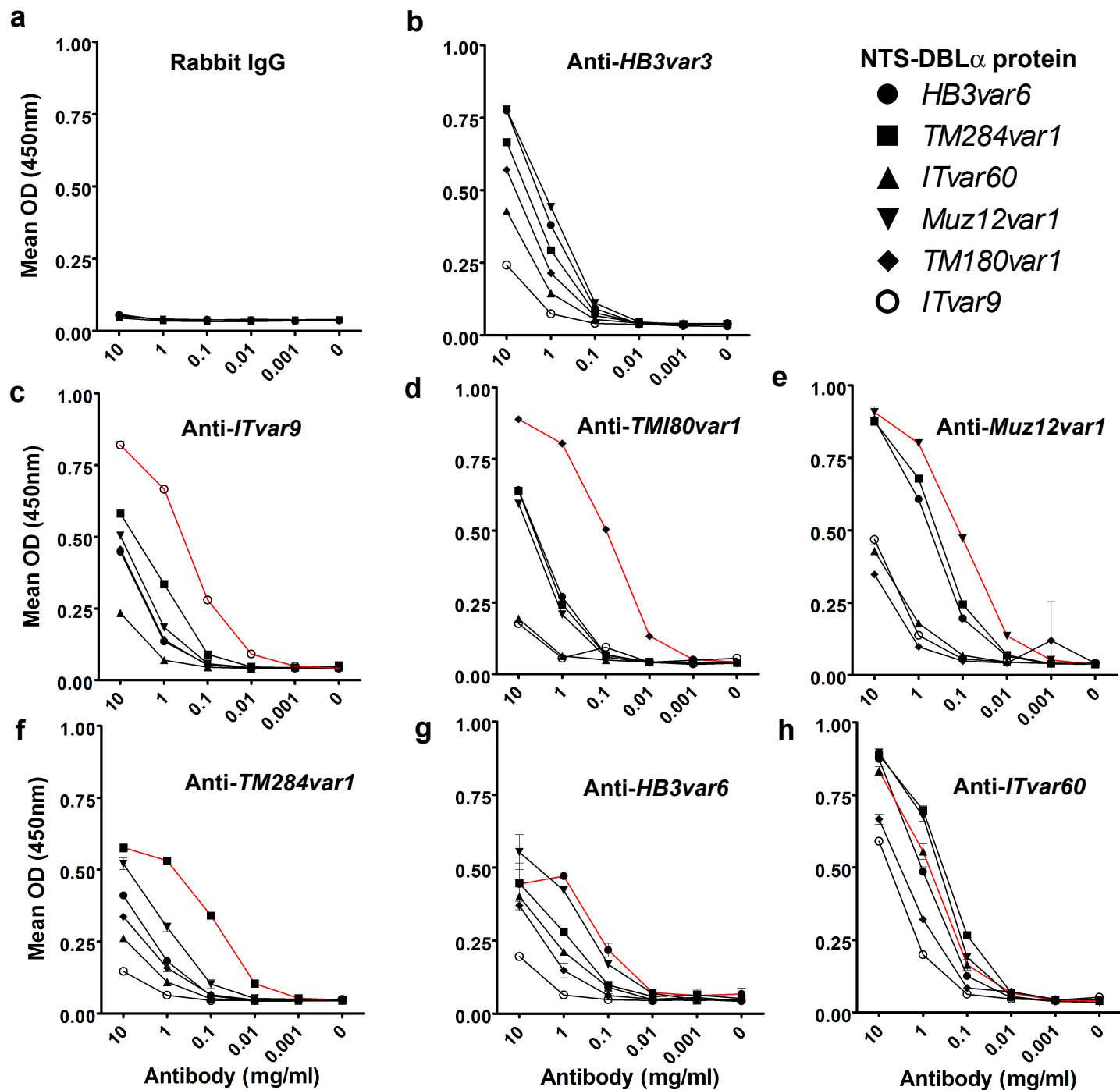

Supplement: Figure S5 — Recognition of recombinant NTS-DBLα domains by homologous and heterologous antibodies by ELISA. Recombinant NTS-DBLα domains of the PfEMP1 variants encoded by HB3var6, TM284var1, ITvar60, Muz12var1, TM180var1 and ITvar9 were coated at 2 µg/ml and incubated with NTS-DBLα antibodies over a range of concentrations from 0–10 µg/ml. Binding was detected using 1∶10,000 dilution of anti-rabbit IgG-HRP (Sigma). The mean and SD of Optical Density (OD) values from triplicate wells are shown. a) rabbit IgG control, b) anti-HB3var3, c) anti-ITvar9, d) anti-TM180var1, e) anti-Muz12var1, f) anti-TM284var1, g) anti-HB3var6 and h) anti-ITvar60. (PDF) [file ppat.1002665.s005.pdf]

**a**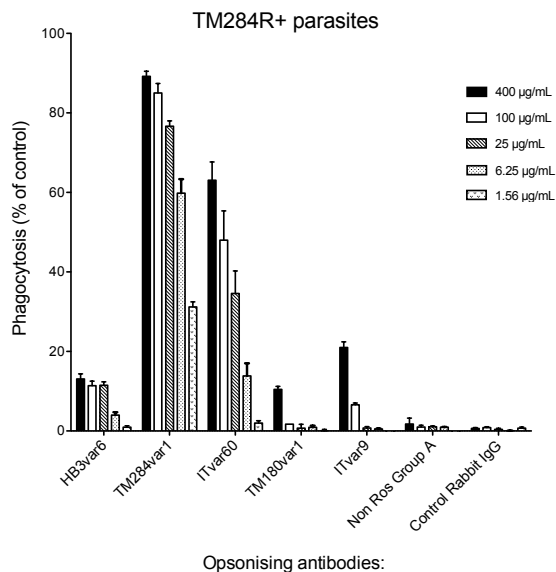**b**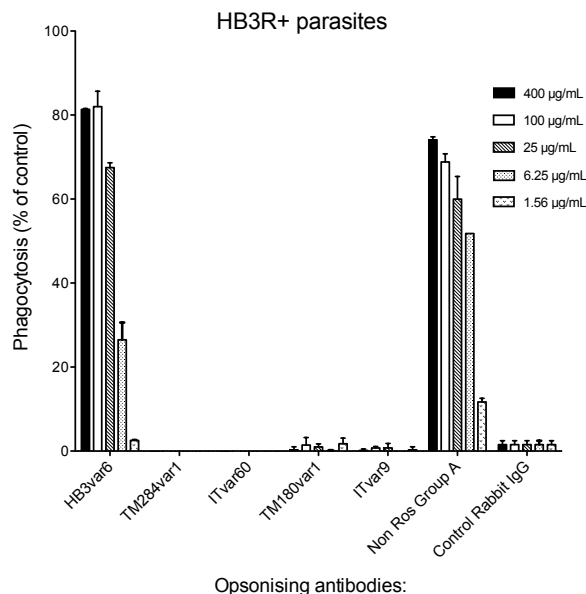**c**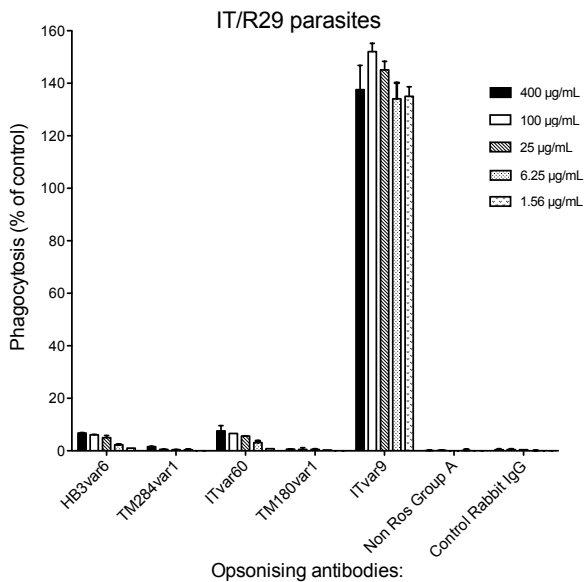**d**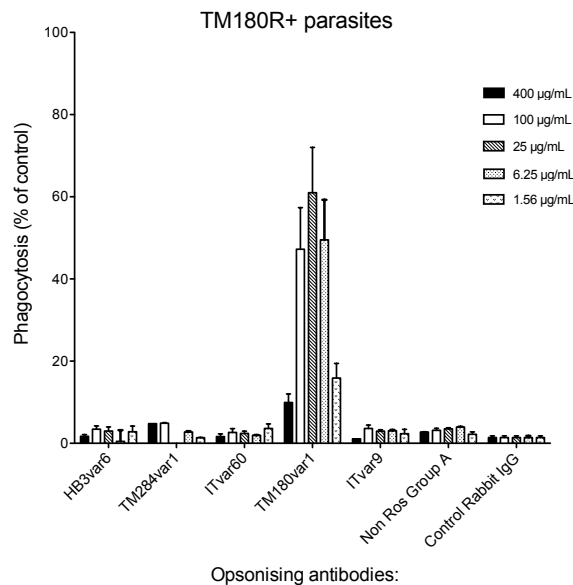

Supplement: Figure S6 — Opsonisation and induction of phagocytosis by polyclonal PfEMP1 antibodies. IEs were stained with ethidium bromide and opsonised with PfEMP1 antibodies over a range of concentrations (1.56–400 µg/ml) before incubation with the monocytic cell line Thp-1. Thp-1 cells that phagocytosed IEs were detected by flow cytometry. a) parasite strain TM284R+, b) parasite strain HB3R+, c) parasite strain IT/R29, d) parasite strain TM180R+. Data are shown as percentage of the positive control opsonised with a rabbit anti-human erythrocyte antibody (ABCAM ab34858 at 90 µg/ml). The “Non Ros Group A” negative control consists of antibodies to HB3var3, a PfEMP1 variant that is not involved in rosetting. HB3R+ parasite culture contains a subpopulation of non-rosetting IEs expressing HB3var3 (see Table S1) which explains why phagocytosis was induced in this case. The “Control Rabbit IgG” is a negative control consisting of IgG from a non-immunized rabbit. (PDF) [file ppat.1002665.s006.pdf]
