# Supplementary material for: Induction of Strain-Transcending Antibodies Against Group A PfEMP1 Surface Antigens from Virulent Malaria Parasites
Source: PLoS Pathog. 2012 Apr 19;8(4):e1002665. doi: 10.1371/journal.ppat.1002665 (PMC3330128; doi:10.1371/journal.ppat.1002665)
Supplement: Figure S3 — Homologous and heterologous polyclonal antibodies to PfEMP1 recognize IgM-positive IEs. a) Parasite strain TM284R+ was stained in a live cell IFA with a mixture of rabbit polylconal antibodies to PfEMP1 (homologous or heterologous) at 20 µg/ml and mouse anti-human IgM mAb (Serotec MCA1662 1/500 dilution). Secondary incubation was with a mixture of Alexa Fluor 488 conjugated anti-rabbit IgG (1/1000) and Alexa Fluor 594-conjugated anti-mouse IgG (1/1000). IEs were stained with DAPI (1 µg/ml; scale bar 10 µm). IgM-positive IEs (right column) show punctate/rim surface fluorescence with both homologous antibodies (anti-TM284var1, 2nd row, middle column) and heterologous antibodies (anti-ITvar60, 3rd row, middle column). 94–100% of the PfEMP1 antibody-positive cells were IgM-positive, and 100% of the IgM-positive cells were PfEMP1-antibody positive. At this concentration TM180var1 antibodies give very faint punctate fluorescence on IgM-positive IEs (5th row, middle column) whereas HB3var6 antibodies are negative (4th row, middle column). At higher concentrations (100–400 µg/ml) both TM180var1 and HB3var6 antibodies stain IgM-positive IEs. b) Specificity controls (with parasite strain TM284R+ as above) show that the Alexa 488 conjugated anti-rabbit IgG secondary does not recognise the mouse anti-human IgM mAb (top row) and that the Alexa 594-conjugated anti-mouse IgG does not recognise the rabbit polyclonal antibodies (bottom row). Camera exposure settings and image handling were identical for PfEMP1 antibodies and controls. (PDF) [file ppat.1002665.s003.pdf]

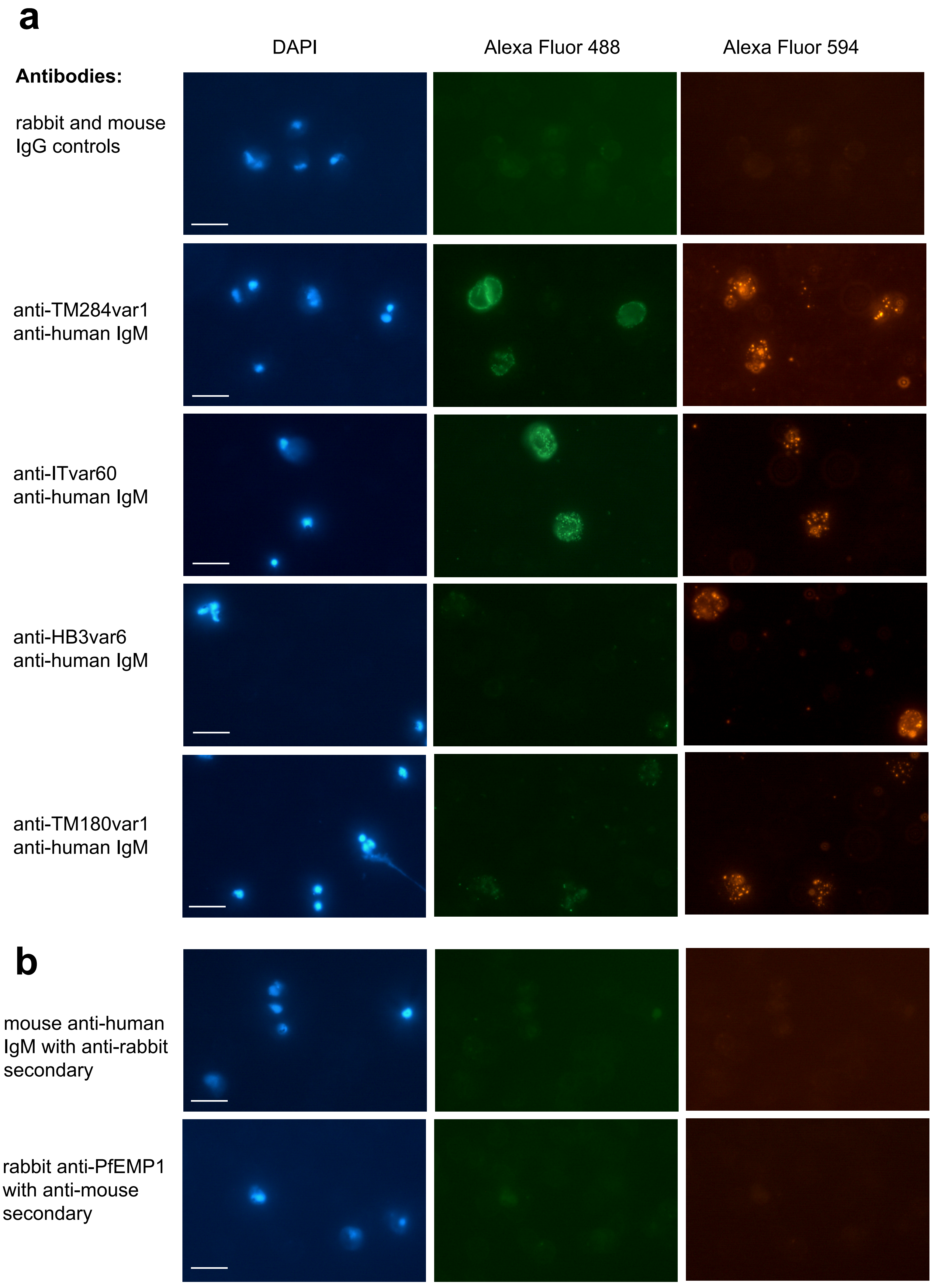
a
DAPI
Alexa Fluor 488
Alexa Fluor 594
Antibodies:
rabbit and mouse IgG controls
anti-TM284var1 anti-human IgM
anti-ITvar60 anti-human IgM
anti-HB3var6 anti-human IgM
anti-TM180var1 anti-human IgM
b
mouse anti-human IgM with anti-rabbit secondary
rabbit anti-PfEMP1 with anti-mouse secondary
