# Supplementary material for: Induction of Strain-Transcending Antibodies Against Group A PfEMP1 Surface Antigens from Virulent Malaria Parasites
Source: PLoS Pathog. 2012 Apr 19;8(4):e1002665. doi: 10.1371/journal.ppat.1002665 (PMC3330128; doi:10.1371/journal.ppat.1002665)
Supplement: Figure S4 — Trypsin-sensitivity of IE surface molecules recognized by heterologous polyclonal PfEMP1 antibodies. a) Flow cytometry of live IEs of P. falciparum strain TM284R+ stained with homologous (anti-TM284var1) and heterologous (anti-ITvar60 and anti-TM180var1) PfEMP1 antibodies (100 µg/ml of total IgG). The negative control was IgG from a non-immunized rabbit (rabbit IgG). IEs were stained with Hoechst and rabbit IgG bound to the surface of IEs was detected with highly cross-absorbed Alex Fluor 488-conjugated anti-rabbit IgG at 1/500 dilution. The IE molecules recognised by PfEMP1 antibodies were sensitive to trypsin (right column) (10 µg/ml trypsin for 5 mins at room temperature (RT), followed by 1 mg/ml of trypsin inhibitor for 5 mins at RT). b) Flow cytometry of live IEs of P. falciparum strain IT/PAR+ stained with homologous (anti-ITvar60) and heterologous (anti-TM284var1 and anti-HB3var6) PfEMP1 antibodies (100 µg/ml of total IgG). Rabbit IgG control and dilutions as above. Trypsin treatment was as above except using 1 mg/ml trypsin. For IT/PAR+ the IE molecules recognised by both homologous and heterologous PfEMP1 antibodies are trypsin-resistant. (PDF) [file ppat.1002665.s004.pdf]

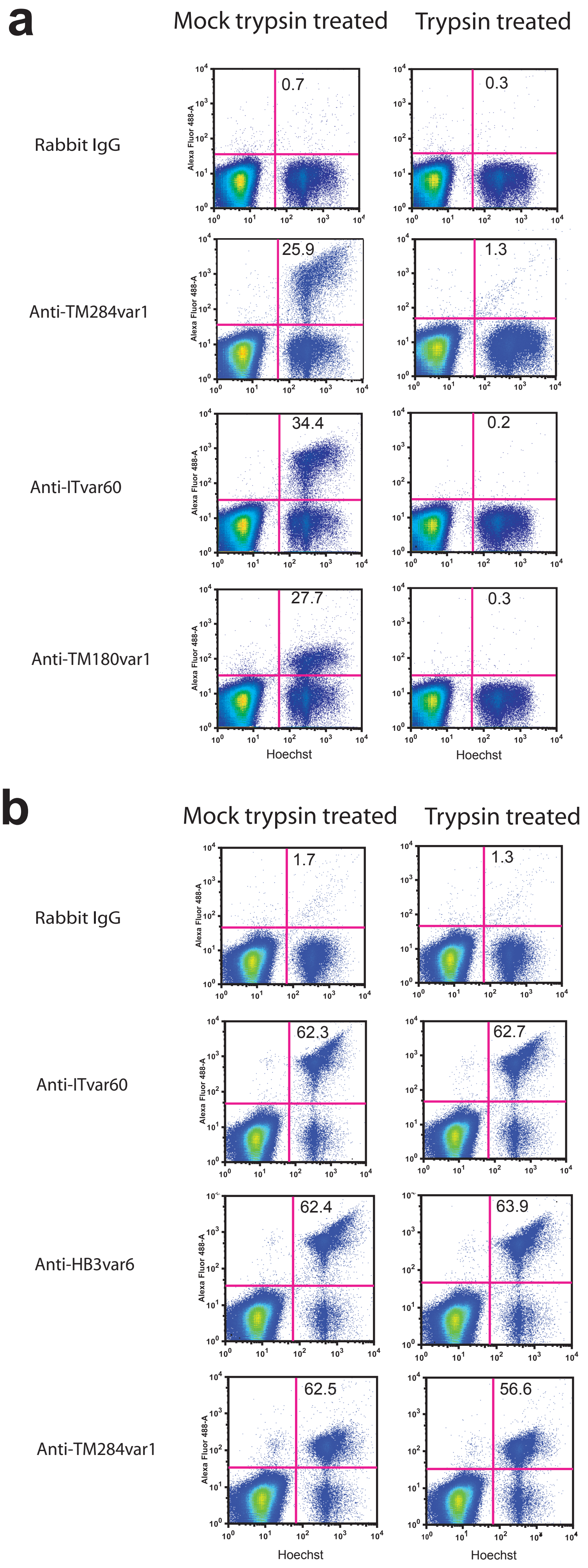
a
Mock trypsin treated
Trypsin treated
Rabbit IgG
0.7
0.3
Anti-TM284var1
25.9
1.3
Anti-ITvar60
34.4
0.2
Anti-TM180var1
27.7
0.3
Alexa Fluor 488-A
Hoechst
b
Mock trypsin treated
Trypsin treated
Rabbit IgG
1.7
1.3
Anti-ITvar60
62.3
62.7
Anti-HB3var6
62.4
63.9
Anti-TM284var1
62.5
56.6
Alexa Fluor 488-A
Hoechst
