# Supplementary material for: Induction of Strain-Transcending Antibodies Against Group A PfEMP1 Surface Antigens from Virulent Malaria Parasites
Source: PLoS Pathog. 2012 Apr 19;8(4):e1002665. doi: 10.1371/journal.ppat.1002665 (PMC3330128; doi:10.1371/journal.ppat.1002665)
Supplement: Table S1 — Identification of a predominant rosette-specific var gene by transcriptional profiling of isogenic rosetting (R+) and non-rosetting (R−) HB3 parasites. (DOC) [file ppat.1002665.s007.doc]

**Table S1.** **Identification of a predominant rosette-specific *var* gene by transcriptional profiling of isogenic rosetting (R+) and non-rosetting (R-) HB3 parasites.**

| HB3R+ Rosette frequency 58% | | | HB3R- Rosette frequency 2% | | |
| --- | --- | --- | --- | --- | --- |
| Number of recombinant plasmids | Gene name | Upstream sequence | Number of recombinant plasmids | Gene name | Upstream sequence |
| **14** | **HB3var6** | **A** | 11 | HB3var29 | C |
| 10 | HB3var3 | A | 7 | HB3var27 | B |
| 3 | HB3var31 | C | 5 | HB3var34 | C |
| 2 | HB3var29 | C | 2 | HB3var28 | C |
| 2 | HB3var34 | C | 2 | HB3var51 | C |
| 2 | HB3var27 | B | 2 | HB3var17 | B |
| 1 | HB3var1CSA | A | 1 | HB3var3 | A |
| 1 | HB3var7 | B | **1** | **HB3var6** | **A** |
| 1 | HB3var24 | B | 1 | HB3var11 | B |
|  |  |  | 1 | HB3var14 | B |
|  |  |  | 1 | HB3var19 | B |

To identify the predominant rosette-specific PfEMP1 variant, the *var* gene transcriptional profiles of isogenic rosetting (R+) and non-rosetting (R-) parasites were compared. RNA was extracted from late ring stage parasites and *var* gene transcription assessed by reverse-transcriptase (RT)-PCR with degenerate primers to DBLα [1,2]. The RT-PCR products were cloned by TA cloning (Invitrogen), and 40 colonies picked for mini-prep DNA extraction and sequencing [3]. From the HB3R+ line (rosette frequency 58%), 36 recombinant plasmids with *var* gene inserts were obtained, and the most common sequence (39% of clones) was the group A *var* gene *HB3var6* (shown in **bold**). This gene was found in only one out of 34 *var* gene inserts sequenced from the HB3R- line (rosette frequency 2%), whereas several group B and C *var* genes were detected commonly in the non-rosetting line. Another group A *var* gene was also common in the HB3R+ line (*HB3var3*, 10/36 clones) and rare in the HB3R- line (1/34 clones). A second independent rosette selection starting from a different cryostabilate of HB3 parasites showed *HB3var6* in 5/16 clones from R+ parasites and 0/15 clones from R- parasites, whereas *HB3var3* was not detected in either R+ or R- populations. These data show that the predominant *var* gene transcribed in HB3 rosetting parasites is *HB3var6.* The same procedure was followed for other *P. falciparum* rosetting strains, with at least two independent selections and RT-PCRs indicating a predominant *var* gene in each case. This semi-quantitative method is reliable for identification of single predominant *var* genes responsible for an adhesion phenotype such as rosetting [4,5], although it does not give accurate quantitation of proportions of different transcripts and may not be suitable in cases where multiple different variants encode a binding phenotype (eg. CD36-binding). In addition to the predominant gene, parasite populations show transcription of numerous other *var* genes at lower frequencies because *var* gene switching occurs constantly in *in vitro* cultures. In particular, *P. falciparum* strains rapidly switch away from Group A *var* genes towards Group B and C types in *in vitro* cultures [6]. Therefore despite regular selection for rosetting, the population remains heterogeneous and the rosetting phenotype is rapidly lost unless selection is continued regularly (2-3 times a week).

**References**

1. Taylor HM, Kyes SA, Harris D, Kriek N, Newbold CI (2000) A study of *var* gene transcription in vitro using universal *var* gene primers. Mol Biochem Parasitol 105: 13-23.

2. Bull PC, Berriman M, Kyes S, Quail MA, Hall N, et al. (2005) *Plasmodium falciparum* Variant Surface Antigen Expression Patterns during Malaria. PLoS Pathog 1: e26.

3. Kyriacou HM, Stone GN, Challis RJ, Raza A, Lyke KE, et al. (2006) Differential *var* gene transcription in *Plasmodium falciparum* isolates from patients with cerebral malaria compared to hyperparasitaemia. Mol Biochem Parasitol 150: 211-218.

4. Rowe JA, Moulds JM, Newbold CI, Miller LH (1997) *P. falciparum* rosetting mediated by a parasite-variant erythrocyte membrane protein and complement-receptor 1. Nature 388: 292-295.

5. Claessens A, Ghumra A, Gupta AP, Mok S, Bozdech Z, et al. (2011) Design of a variant surface antigen-supplemented microarray chip for whole transcriptome analysis of multiple *Plasmodium falciparum* cytoadherent strains, and identification of strain-transcendent *rif* and *stevor* genes. Malar J 10: 180.

6. Peters JM, Fowler EV, Krause DR, Cheng Q, Gatton ML (2007) Differential changes in P*lasmodium falciparum var* transcription during adaptation to culture. J Infect Dis 195: 748-755.
