# Supplementary material for: Induction of Strain-Transcending Antibodies Against Group A PfEMP1 Surface Antigens from Virulent Malaria Parasites
Source: PLoS Pathog. 2012 Apr 19;8(4):e1002665. doi: 10.1371/journal.ppat.1002665 (PMC3330128; doi:10.1371/journal.ppat.1002665)
Supplement: Table S2 — Pair-wise amino acid identities for NTS-DBLα, CIDR1 and DBLγ from rosetting PfEMP1 variants. (DOC) [file ppat.1002665.s008.doc]

**Table S2. Pair-wise amino acid identities for NTS-DBLα, CIDR1 and DBLγ from rosetting PfEMP1 variants^a^**

|  | HB3  var6 | TM284  var1 | IT  var60 | Muz12  var1 | TM180  var1 | IT  var9 | PaloAlto  varO | 3D7  PF13_0003 |
| --- | --- | --- | --- | --- | --- | --- | --- | --- |
| **Pair-wise amino acid identities for NTS-DBLα** | | | | | | | | |
| HB3var6 | 100 | 50.8^b^ | 51.3 | 61.0 | 41.8 | 46.7 | 47.3 | 49.1 |
| TM284var1 |  | 100 | 62.6 | 47.0 | 41.4 | 58.7 | 54.1 | 47.5 |
| ITvar60 |  |  | 100 | 43.6 | 38.9 | 53.8 | 55.3 | 52.5 |
| Muz12var1 |  |  |  | 100 | 43.0 | 44.0 | 47.3 | 49.0 |
| TM180var1 |  |  |  |  | 100 | 40.8 | 42.6 | 39.3 |
| ITvar9 |  |  |  |  |  | 100 | 61.6 | 53.9 |
| PA varO |  |  |  |  |  |  | 100 | 59.6 |
| 3D7 PF13 |  |  |  |  |  |  |  | 100 |
|  |  |  |  |  |  |  |  |  |
| **Pair-wise amino acid identities for CIDR1** | | | | | | | | |
| HB3var6 | 100 | 45.1 | 48.6 | 81.1 | 24.9 | 34.4 | 40.4 | 60.7 |
| TM284var1 |  | 100 | 82.2 | 44.6 | 23.2 | 37.2 | 37.9 | 45.6 |
| ITvar60 |  |  | 100 | 46.7 | 25.3 | 40.0 | 38.6 | 48.4 |
| Muz12var1 |  |  |  | 100 | 24.6 | 35.1 | 37.9 | 57.2 |
| TM180var1 |  |  |  |  | 100 | 21.4 | 20.7 | 24.6 |
| ITvar9 |  |  |  |  |  | 100 | 35.8 | 35.4 |
| PA varO |  |  |  |  |  |  | 100 | 33.7 |
| 3D7 PF13 |  |  |  |  |  |  |  | 100 |
|  |  |  |  |  |  |  |  |  |
| **Pair-wise amino acid identities for DBLγ** | | | | | | | | |
| HB3var6 | 100 | 38.3 | 38.1 | 38.9 | 46.6 | 34.1 | 40.9 | 49.4 |
| TM284var1 |  | 100 | 56.5 | 38.3 | 36.3 | 29.3 | 40.1 | 37.8 |
| ITvar60 |  |  | 100 | 38.8 | 34.9 | 27.9 | 41.0 | 34.5 |
| Muz12var1 |  |  |  | 100 | 45.4 | 31.1 | 43.8 | 40.6 |
| TM180var1 |  |  |  |  | 100 | 28.8 | 49.7 | 35.3 |
| ITvar9 |  |  |  |  |  | 100 | 29.9 | 33.3 |
| PA varO |  |  |  |  |  |  | 100 | 37.3 |
| 3D7 PF13 |  |  |  |  |  |  |  | 100 |
|  |  |  |  |  |  |  |  |  |

^a^Rosetting variants described in this work plus ITvar9 [1], Palo Alto var O [2] and PF13_0003 [3]. *ITvar9, Palo Alto varO* and *PF13_0003* encode PfEMP1 variants expressed by IgM-negative rosetting strains.

^b^Pair-wise amino acid identities between the IgM-positive rosetting strains shown in red

**References.**

1. Rowe JA, Moulds JM, Newbold CI, Miller LH (1997) *P. falciparum* rosetting mediated by a parasite-variant erythrocyte membrane protein and complement-receptor 1. Nature 388: 292-295.

2. Vigan-Womas I, Guillotte M, Le Scanf C, Igonet S, Petres S, et al. (2008) An in vivo and in vitro model of *Plasmodium falciparum* rosetting and autoagglutination mediated by *varO*, a group A *var* gene encoding a frequent serotype. Infect Immun 76: 5565-5580.

3. Vigan-Womas I, Guillotte M, Juillerat A, Vallieres C, Lewit-Bentley A, et al. (2011) Allelic diversity of the *Plasmodium falciparum* erythrocyte membrane protein 1 entails variant-specific red cell surface epitopes. PLoS One 6: e16544.
