# Supplementary material for: Induction of Strain-Transcending Antibodies Against Group A PfEMP1 Surface Antigens from Virulent Malaria Parasites
Source: PLoS Pathog. 2012 Apr 19;8(4):e1002665. doi: 10.1371/journal.ppat.1002665 (PMC3330128; doi:10.1371/journal.ppat.1002665)
Supplement: Table S3 — Pair-wise amino acid identities for DBLε from rosetting PfEMP1 variants. (DOC) [file ppat.1002665.s009.doc]

**Table S3. Pair-wise amino acid identities for DBLε from rosetting PfEMP1 variants**

|  | HB3  var6  d4^a^ | HB3  var6  d6^b^ | TM284  var1  d3^c^ | TM284  var1  d5^d^ | IT  var60 d4^a^ | IT  var60 d5^d^ | IT  var9 d3^c^ | Palo Alto varO  d5^d^ |
| --- | --- | --- | --- | --- | --- | --- | --- | --- |
| HB3var6 d4^a^ | 100 | 28.6^e^ | 43.5 | 23.2 | 23.7 | 22.0 | 24.5 | 27.3 |
| HB3var6 d6^b^ |  | 100 | 30.5 | 29.9 | 30.5 | 28.6 | 38.8 | 38.5 |
| TM284var1 d3^c^ |  |  | 100 | 25.3 | 22.7 | 26.1 | 26.3 | 28.6 |
| TM284var1 d5^d^ |  |  |  | 100 | 23.7 | 41.7 | 28.9 | 32.1 |
| ITvar60 d4^a^ |  |  |  |  | 100 | 24.3 | 32.1 | 29.6 |
| ITvar60 d5^d^ |  |  |  |  |  | 100 | 27.6 | 31.5 |
| ITvar9 d3^c^ |  |  |  |  |  |  | 100 | 30.9 |
| PA varO d5^d^ |  |  |  |  |  |  |  | 100 |

^a^d4: 4th DBL domain from the N-terminus

^b^d6: 6th DBL domain from the N-terminus

^c^d3: 3rd DBL domain from the N-terminus

^d^d5: 5th DBL domain from the N-terminus

^e^Pair-wise amino acid identities between the IgM-positive rosetting strains shown in red
