# Supplementary material for: Induction of Strain-Transcending Antibodies Against Group A PfEMP1 Surface Antigens from Virulent Malaria Parasites
Source: PLoS Pathog. 2012 Apr 19;8(4):e1002665. doi: 10.1371/journal.ppat.1002665 (PMC3330128; doi:10.1371/journal.ppat.1002665)
Supplement: Table S4 — Pair-wise amino acid identities for DBLζ from rosetting PfEMP1 variants. (DOC) [file ppat.1002665.s010.doc]

**Table S4. Pair-wise amino acid identities for DBLζ from rosetting PfEMP1 variants**

|  | HB3var6 | TM284var1 | ITvar60 | Palo Alto varO |
| --- | --- | --- | --- | --- |
| HB3var6 | 100 | 42.7^a^ | 37.1 | 36.1 |
| TM284var1 |  | 100 | 35.6 | 35.0 |
| ITvar60 |  |  | 100 | 36.8 |
| Palo Alto varO |  |  |  | 100 |

^a^Pair-wise amino acid identities between the IgM-positive rosetting strains shown in red
