# Supplementary material for: Induction of Strain-Transcending Antibodies Against Group A PfEMP1 Surface Antigens from Virulent Malaria Parasites
Source: PLoS Pathog. 2012 Apr 19;8(4):e1002665. doi: 10.1371/journal.ppat.1002665 (PMC3330128; doi:10.1371/journal.ppat.1002665)
Supplement: Table S5 — Pair-wise amino acid identities for DBLβ from rosetting PfEMP1 variants. (DOC) [file ppat.1002665.s011.doc]

**Table S5. Pair-wise amino acid identities for DBLβ from rosetting PfEMP1** variants

|  | Muz12var1 | TM180var1 | Palo Alto varO | 3D7 PF13_0003 |
| --- | --- | --- | --- | --- |
| Muz12var1 | 100 | 46.8 | 46.9 | 46.4 |
| TM180var1 |  | 100 | 49.3 | 49.2 |
| Palo Alto varO |  |  | 100 | 46.9 |
| 3D7 PF13_0003 |  |  |  | 100 |
