# Supplementary material for: Induction of Strain-Transcending Antibodies Against Group A PfEMP1 Surface Antigens from Virulent Malaria Parasites
Source: PLoS Pathog. 2012 Apr 19;8(4):e1002665. doi: 10.1371/journal.ppat.1002665 (PMC3330128; doi:10.1371/journal.ppat.1002665)
Supplement: Table S7 — Pair-wise amino acid identities for CIDR2 from rosetting PfEMP1 variants. (DOC) [file ppat.1002665.s013.doc]

**Table S7.** Pair-wise amino acid identities for CIDR2 from rosetting PfEMP1 variants

|  | HB3var6 | Muz12var1 | TM180var1 | ITvar9 | Palo Alto varO | 3D7  PF13_0003 |
| --- | --- | --- | --- | --- | --- | --- |
| HB3var6 | 100 | 28.1 | 30.9 | 26.7 | 53.0 | 32.3 |
| Muz12var1 |  | 100 | 45.3 | 50.2 | 33.3 | 41.8 |
| TM180var1 |  |  | 100 | 37.9 | 34.4 | 48.1 |
| ITvar9 |  |  |  | 100 | 29.8 | 36.8 |
| PA varO |  |  |  |  | 100 | 35.1 |
| 3D7 PF13 |  |  |  |  |  | 100 |
