# Supplementary material for: Induction of Strain-Transcending Antibodies Against Group A PfEMP1 Surface Antigens from Virulent Malaria Parasites
Source: PLoS Pathog. 2012 Apr 19;8(4):e1002665. doi: 10.1371/journal.ppat.1002665 (PMC3330128; doi:10.1371/journal.ppat.1002665)
Supplement: Table S8 — Summary of PfEMP1 antibody activity against homologous parasite strains. (DOC) [file ppat.1002665.s014.doc]

**Table S8. Summary of PfEMP1 antibody activity against homologous parasite strains**

| Antibodies to: | Surface reactivity  (end titre^a^) | % positive cells matches rosette frequency^b^ | Trypsin- sensitive surface reactivity^c^ | Rosette inhibition  (IC50^d^) | Phagoctyosis ~50% of positive control^e^ |
| --- | --- | --- | --- | --- | --- |
| HB3var6 | + (1.56) | + | Yes | + (1.00) | 6.25 |
| TM284var1 | + (0.10) | + | Yes | + (0.08) | 6.25 |
| ITvar60 | + (0.10) | + | No | + (0.08) | <1.56 |
| Muz12var1 | + (0.02) | + | Yes | + (8.00) | ND |
| TM180var1 | + (0.10) | + | Yes | - | 6.25 |
| ITvar9^f^ | + (0.02) | + | Yes | + (0.04) | <1.56 |

^a^ The end titre was defined as the lowest concentration of antibody (in μg/ml of total IgG) giving surface staining of more than 50% of the positive subpopulation by flow cytometry.

^b^ + indicates that the percentage of infected erythrocytes showing punctate surface fluorescence in live cell IFA matched the rosette frequency of the culture to within 10% in at least three separate experiments. Rosette frequency varied between 30% and 75% in different experiments due to *var* gene switching and frequency of rosette selection.

^c^ Surface reactivity abolished by treatment of live infected erythrocytes with low concentration trypsin (10 μg/ml for 5 mins at room temperature).

^d^ IC50: concentration of total IgG in μg/ml giving 50% rosette inhibition.

^e^ In μg/ml of total IgG.

^f^ Data from reference 13 (Ghumra et al, 2011 PLoS One 6:e16414.)

ND: not done.
