# Supplementary material for: Induction of Strain-Transcending Antibodies Against Group A PfEMP1 Surface Antigens from Virulent Malaria Parasites
Source: PLoS Pathog. 2012 Apr 19;8(4):e1002665. doi: 10.1371/journal.ppat.1002665 (PMC3330128; doi:10.1371/journal.ppat.1002665)
Supplement: Table S9 — IgM phenotype of HB3R+ and Muz12R+ parasites. (DOC) [file ppat.1002665.s015.doc]

**Table S9. IgM phenotype of HB3R+ and Muz12R+ parasites**

| Parasite strain | Rosette frequency (%) | Frequency of IgM positive infected erythrocytes by live cell IFA (%) |
| --- | --- | --- |
| HB3R+ expt 1 | 64 | 68^a^ |
| HB3R+ expt 2 | 57 | 51^a^ |
| HB3R+ expt 3 | 59 | 53^a^ |
| Muz12R+ expt 1 | 46 | 5 |
| Muz12R+ expt 2 | 59 | 0 |
| Muz12R+ expt 3 | 46 | 5 |

^a^ IgM positive infected erythrocytes in wet preparations are seen in rosettes
