# Supplementary material for: Induction of Strain-Transcending Antibodies Against Group A PfEMP1 Surface Antigens from Virulent Malaria Parasites
Source: PLoS Pathog. 2012 Apr 19;8(4):e1002665. doi: 10.1371/journal.ppat.1002665 (PMC3330128; doi:10.1371/journal.ppat.1002665)
Supplement: Text S1 — Western blots to detect ITvar60 PfEMP1. (DOC) [file ppat.1002665.s016.doc]

**Text S1. Western blots to detect ITvar60 PfEMP1.**

To investigate whether the ITvar60 PfEMP1 variant is trypsin-resistant, or whether the ITvar60 antibodies detect other (non-PfEMP1, trypsin-resistant) molecules, we carried out western blots of detergent extracts of IT/PAR+ parasites probed either with a monoclonal antibody ( mAb 6H1) to the acidic terminal sequence of PfEMP1 [1] or with the rabbit polyclonal antibodies to ITvar60 NTS-DBLα. PfEMP1 can be detected on western blots as a high molecular weight (>250kDa), Triton-X-100 insoluble, SDS-soluble protein [2]. Using the 6H1 mAb we found that the PfEMP1 variant from IT/PAR+ parasites was not cleaved by treatment of whole infected erythrocytes with 1 mg/ml of trypsin (Figure S2a). The effectiveness of trypsin was shown with extracts from parasite strain IT/R29 that show the trypsin-sensitivity more usually seen with PfEMP1 variants (Figure S2b). The 6H1 mAb also recognizes high molecular weight bands present in normal RBC in some donors (Fig S2a) but not others (Fig S2b). The trypsin-resistance of the PfEMP1 variant from IT/PAR+ parasites is unusual but not unprecedented, as trypsin-resistance has been described previously for the pregnancy-malaria associated PfEMP1 variant var2CSA [3], which may be due to its compact, globular structure [4]. A repeated blot of IT/PAR+ triton-insoluble, SDS-soluble extracts with mAb 6H1 showed clear evidence for two high molecular weight proteins (Fig 2c). The upper band (black arrow) may represent the ITvar60 variant (predicted molecular weight 315 kDa), while the lower band (grey arrow) may represent another commonly expressed variant in this parasite line, such as the previously identified FCR3S1.2 variant [5] (predicted molecular weight 253 kDa). Poor resolution of very high molecular weight proteins and lack of specific high molecular weight markers makes it difficult to estimate precise molecular weights for these proteins. The lower 6H1-specific band may also be present in Figure S2a, but this is difficult to discern due to the red cell background bands present in this blot.

Western blotting with the rabbit polyclonal antibodies to ITvar60 showed that these antibodies also recognise two main high molecular weight, Triton-X-100 insoluble, SDS-soluble, trypsin-resistant proteins (Figure S2c). Therefore the ITvar60 antibodies are detecting PfEMP1-like high molecular weight proteins. No other parasite-specific bands were detected by the ITvar60 antibodies on either reduced or non-reduced gels, making it unlikely that these antibodies are recognizing proteins other than PfEMP1 on the infected erythrocyte surface. Triton-X-100 soluble bands at approximately 70 and 50 kDa (Figure S2d), and 40, 30 and 20 kDa (not shown) were detected by the ITvar60 antibodies, however these bands were not parasite-specific, as they were also present in detergent extracts from uninfected red cells (Figure S2d). Similar Triton-soluble red cell proteins of 20-70 kDa were also seen when blots were probed with non-immunised rabbit IgG (not shown) therefore they probably represent non-specific recognition of human red cell proteins by rabbit IgG, and cannot explain the specific recognition of infected erythrocytes shown by the antibodies generated to PfEMP1 domains.

**Methods.**

**Western blotting.** Cells were solubilized in 150 mM NaCl, 5 mM EDTA, 50 mM Tris pH 8.0, 1% (w/v) Triton X-100 with protease inhibitors to generate the Triton X-100-soluble fraction (Tx). Triton X-100-insoluble pellets were extracted with the above buffer supplemented with 2% SDS to produce the Triton X-100-insoluble, SDS-soluble (SDS) fraction. For each fraction, 3-5μg of parasite proteins were heated to 70°C under reducing (plus 10mM DTT) or non-reducing conditions and electrophoresed on 3-8% Tris-Acetate gels as per the manufacturers instructions (Invitrogen). Proteins were then transferred onto PVDF membrane (Millipore). For experiments with the mouse mAb 6H1 [1], membranes were probed with 1/1000 dilution of mAb 6H1 followed by 1/1000 dilution of HRP-conjugated sheep-anti-mouse IgG (Chemicon International). The signal was developed with ECL plus western blotting detection reagents as described by the manufacturer (Amerhsam Biosciences). For experiments with the rabbit polyclonal NTS-DBLα antibodies, membranes were incubated with 1:15,000 dilution in PBS overnight at 4°C in 2.5% skim milk. The following day, the membranes were washed 3x 5 minutes with PBS-Tween 0.5%. The primary antibodies were detected by goat-anti-rabbit HRP secondary at 1:20,000 (Sigma) and again washed 3x 5 minutes in PBS-Tween 0.5%. The blots were then incubated for 5 minutes with Immobilon Western Blot chemiluminescent HRP substrate (Millipore) and exposed onto Hyperfilm ECL (GE).

**References**

1. Duffy MF, Brown GV, Basuki W, Krejany EO, Noviyanti R, et al. (2002) Transcription of multiple var genes by individual, trophozoite-stage *Plasmodium falciparum* cells expressing a chondroitin sulphate A binding phenotype. Mol Microbiol 43: 1285-1293.

2. Leech JH, Barnwell JW, Miller LH, Howard RJ (1984) Identification of a strain-specific malarial antigen exposed on the surface of *Plasmodium falciparum* infected erythrocytes. J Exp Med 159: 1567-1575.

3. Nielsen MA, Resende M, Alifrangis M, Turner L, Hviid L, et al. (2007) *Plasmodium falciparum*: VAR2CSA expressed during pregnancy-associated malaria is partially resistant to proteolytic cleavage by trypsin. Exp Parasitol 117: 1-8.

4. Srivastava A, Gangnard S, Round A, Dechavanne S, Juillerat A, et al. (2010) Full-length extracellular region of the var2CSA variant of PfEMP1 is required for specific, high-affinity binding to CSA. Proc Natl Acad Sci U S A 107: 4884-4889.

5. Chen Q, Barragan A, Fernandez V, Sundstrom A, Schlichtherle M, et al. (1998) Identification of *Plasmodium falciparum* erythrocyte membrane protein 1 (PfEMP1) as the rosetting ligand of the malaria parasite *P. falciparum*. J Exp Med 187: 15-23.
