# Supplementary material for: Induction of Strain-Transcending Antibodies Against Group A PfEMP1 Surface Antigens from Virulent Malaria Parasites
Source: PLoS Pathog. 2012 Apr 19;8(4):e1002665. doi: 10.1371/journal.ppat.1002665 (PMC3330128; doi:10.1371/journal.ppat.1002665)
Supplement: Text S2 — Reactivity of PfEMP1 antibodies with recombinant NTS-DBLα recombinant proteins by ELISA. (DOC) [file ppat.1002665.s017.doc]

**Text S2. Reactivity of PfEMP1 antibodies with recombinant NTS-DBLα recombinant proteins by ELISA**

We examined whether the pattern of surface reactivity with homologous and heterologous live infected erythrocytes shown in Figure 4b was also seen when each antibody was tested in an ELISA against the panel of NTS-DBLα recombinant proteins used for immunization. Non-immunized rabbit IgG was negative by ELISA (Figure S5a), but antibodies to the negative control non-rosetting Group A PfEMP1 variant HB3var3 showed positive O.D. readings against all NTS-DBLα recombinant proteins (Figure S5b). The anti-HB3var3 antibodies did not recognise infected erythrocyte surface molecules from any of the rosetting strains (Figure 4b), hence ELISA results clearly differ from surface reactivity in this case. For the antibodies showing predominantly variant-specific, strain-specific surface reactivity such as anti-ITvar9 and anti-TM180var1 (Figure 4b), in ELISA the homologous antigen gave the highest O.D. values, however other NTS-DBLα recombinant proteins were also recognised (Figure S5c and S5d). Therefore in these examples also, the ELISA results do not correspond with surface reactivity. For antibodies to Muz12var1 and TM284var1, which both show heterologous surface reactivity against two other parasite strains (Figure 4b), ELISA shows highest values for the homologous antigen (Figure S5e and S5f), however, heterologous NTS-DBLα recombinant proteins were also well-recognised in a pattern that did not correspond with surface reactivity. For antibodies to HB3var6 and ITvar60, multiple NTS-DBLα recombinant proteins were recognised in ELISA (Figure S5g and S5h), and again these results do not correspond with surface reactivity. Taken together, these data confirm the results of Vigan-Womas *et al* [1], which show that ELISA results with DBL recombinant proteins do not predict surface reactivity with live infected erythrocytes.

**Reference.**

**1. Vigan-Womas I, Guillotte M, Juillerat A, Vallieres C, Lewit-Bentley A, et al.** (2011) Allelic diversity of the *Plasmodium falciparum* erythrocyte membrane protein 1 entails variant-specific red cell surface epitopes. PLoS One 6: e16544.
